# Classification of SARS-CoV-2 sequences as recombinants via a pre-trained CNN and identification of a mathematical signature relative to recombinant feature at Spike, via interpretability

**DOI:** 10.1371/journal.pone.0309391

**Published:** 2024-08-26

**Authors:** Ana Guerrero-Tamayo, Borja Sanz Urquijo, Isabel Olivares, María-Dolores Moragues Tosantos, Concepción Casado, Iker Pastor-López

**Affiliations:** 1 Faculty of Engineering, University of Deusto, Bilbao, Biscay, Spain; 2 National Microbiology Center (NMC), Instituto de Salud Carlos III (ISCIII), Majadahonda, Madrid, Spain; 3 Faculty of Medicine and Nursing University of the Basque Country UPV/EHU, Leioa, Biscay, Spain; UPES: University of Petroleum and Energy Studies, INDIA

## Abstract

The global impact of the SARS-CoV-2 pandemic has underscored the need for a deeper understanding of viral evolution to anticipate new viruses or variants. Genetic recombination is a fundamental mechanism in viral evolution, yet it remains poorly understood. In this study, we conducted a comprehensive research on the genetic regions associated with genetic recombination features in SARS-CoV-2. With this aim, we implemented a two-phase transfer learning approach using genomic spectrograms of complete SARS-CoV-2 sequences. In the first phase, we utilized a pre-trained VGG-16 model with genomic spectrograms of HIV-1, and in the second phase, we applied HIV-1 VGG-16 model to SARS-CoV-2 spectrograms. The identification of key recombination hot zones was achieved using the Grad-CAM interpretability tool, and the results were analyzed by mathematical and image processing techniques. Our findings unequivocally identify the SARS-CoV-2 Spike protein (S protein) as the pivotal region in the genetic recombination feature. For non-recombinant sequences, the relevant frequencies clustered around 1/6 and 1/12. In recombinant sequences, the sharp prominence of the main hot zone in the Spike protein prominently indicated a frequency of 1/6. These findings suggest that in the arithmetic series, every 6 nucleotides (two triplets) in S may encode crucial information, potentially concealing essential details about viral characteristics, in this case, recombinant feature of a SARS-CoV-2 genetic sequence. This insight further underscores the potential presence of multifaceted information within the genome, including mathematical signatures that define an organism’s unique attributes.

## 1 Introduction

The evolution of viruses poses a significant challenge in pandemic control. Two main mechanisms are responsible for the high rate of viral evolution: mutation and genetic recombination. Both mechanisms occur with great frequency during viral replication [1, 2]. Mutations introduce random errors into the genetic material of a virus, resulting in genetic variants of the same virus [3]. Genetic recombination is the exchange of genetic information between two viral genomes of the same or different viruses, resulting in a new hybrid genome [4, 5]. Both processes can generate variants that hinder the prevention and treatment of infectious diseases. Recombination can occur in many RNA viruses, having been detected with high frequency in picornaviruses [6], coronaviruses [7, 8], and retroviruses [9]. Recombinant viruses represent an enigma in the realm of infectious diseases, as their consequences can vary widely. In some instances, genetic recombination may result in viruses showing no significant changes in their behavior or pathogenicity, or remaining relatively harmless. However, in other scenarios, this recombination can lead to the emergence of new viral strains with unique characteristics, such as increased transmission capacity or virulence [10–16]. Even on rare occasions, genetic recombination has been the cause of failure of attenuated virus vaccines [17, 18]. The genetic recombination that occurs among different viruses, as observed in SARS [19] and MERS [20], can have significant implications for viral evolution and its impact on public health [21].

SARS-CoV-2, short for Severe Acute Respiratory Syndrome Coronavirus 2, is a novel coronavirus that emerged in late 2019 and quickly spread to become a global pandemic [22]. This highly contagious virus is responsible for the Coronavirus Disease 2019 (COVID-19), characterized by a range of symptoms from mild respiratory issues to severe pneumonia and, in some cases, death [23]. According to World Health Organization (WHO), as of September 1st, 2023, COVID-19 has caused 770,437,327 confirmed cases, including 6,956,900 deaths [24].

Since the outbreak of the pandemic in January 2020 [25], several variants have emerged with evolutionary characteristics leading to either increased transmissibility or enhanced vaccine evasion. Among the Variants of Concern (VOCs), Alpha (B.1.1.7) stood out, reaching its zenith around April 2021 [26]. Another VOC of significant impact was Delta (B.1.617.2), reaching its peak around September 2021 [27, 28]. From early 2022 to the present day, Omicron and its different subvariants (B.1.1.529, BA.1—BA.5, BQ.1, etc.) have dominated the epidemiological landscape [29, 30].

The recombination events detected in SARS-CoV-2 have occurred solely between different lineages of the virus, with no substantial modifications in terms of morbidity, mortality, etc [31]. Most of these recombinants have occurred between co-circulating Omicron sublineages, such as BA.1 (or BA.1.1), and the Delta variant or BA.2 [32]. However, recombination between Omicron and Delta could potentially result in a virus with Omicron’s transmissibility and Delta’s potentially increased risk of severe illness, leading to a new and more concerning scenario for public health [33].

Understanding genetic recombination phenomena holds paramount importance in anticipating and addressing pandemics, as well as in the surveillance and control of the emergence of new viruses or variants [34].

Deep Learning tools can provide new insights into the study of genetic recombination [35, 36] and the anticipation of new pandemics or emerging viruses [37]. By analyzing large genomic datasets and identifying patterns, Deep Learning tools can detect signs of genetic recombination in viruses more quickly than traditional methods. In this way, we can not only reduce response times to emerging viruses but also anticipate their emergence. Likewise, we can unravel the mysteries of the genetic code from new perspectives, such as the search for mathematical patterns within the genome itself.

## 2 Materials and methods

All experiments were ran in this equipment:

- Processing Unit: Intel(R) Core(TM) i7–4770K CPU. 3.5 GHz.
- Installed RAM: 32 GB usable.
- Operative System: Windows 10 Education. Version: 22H2.
- GPU: NVIDIA GeForce RTX 3090. Total memory: 40 GB.

### 2.1 SARS-CoV-2 complete genomic sequences compendium

We downloaded the complete collections of SARS-CoV-2 sequences by variant from the NCBI Virus Database (National Center for Biotechnology Information, Virus Database), in March 2023. Out of a total of 1,541,293 sequences, 1,539,728 were assigned as non-recombinant, and 1,565 recombinant, and their variant distribution are detailed in Tables 1 and 2, respectively.

**Table 1:** Non-recombinant SARS-CoV-2 sequence compendium. Downloaded from the NCBI Virus Database (National Center for Biotechnology Information, Virus Database) [39], the total of 1,539,728 sequences corresponds to an approximate date of March 2023 (Release Date of NCBI Virus Database). The variants are sorted by the approximate date of appearance (data obtained from GISAID Initiative—Tracking of hCoV-19 Variants) [40]. The column “Variant” indicates the WHO Name of the SARS-CoV-2 variant. The column “No.” indicates the total number of downloaded complete sequences. The “Percentage” column indicates the percentage that this total number of complete sequences represents out of the total downloaded sequences.

Non-recombinant SARS-CoV-2 Sequences		
Variant	No.	Percentage
PRE-VOC	218,198	14.17%
ALPHA	198,722	12.91%
BETA	856	0.06%
GAMMA	11,937	0.78%
DELTA	325,285	21.13%
EPSILON	14,781	0.96%
ETA	738	0.05%
IOTA	19,361	1.26%
KAPPA	145	0.01%
LAMBDA	456	0.03%
MU	49	0.00%
THETA	12	0.00%
ZETA	553	0.04%
OMICRON	748,635	48.62%
**TOTAL**	**1,539,728**	**100%**

**Table 2:** Recombinant SARS-CoV-2 sequence compendium. Downloaded from the NCBI Virus Database (National Center for Biotechnology Information, Virus Database) [39], the total of 1,565 sequences corresponds to an approximate date of March 2023 (Release Date of NCBI Virus Database). The nomenclature for recombinant variants begins with an “X” in the Pango nomenclature [41]. The column “Variant” indicates the WHO Name of the SARS-CoV-2 variant. The column “No.” indicates the total number of downloaded complete sequences. The “Percentage” column indicates the percentage that this total number of complete sequences represents out of the total downloaded sequences.

Recombinant SARS-CoV-2 sequences					
Variant	No.	Percentage	Variant	No.	Percentage
XA	2	0.13%	XBB.3	62	3.96%
XAA	47	3.00%	XBB.4	8	0.51%
XAB	2	0.13%	XBD	30	1.92%
XAC	37	2.36%	XBE	32	2.04%
XAD	1	0.06%	XC	227	14.50%
XAE	18	1.15%	XD	5	0.32%
XAF	16	1.02%	XE	256	16.36%
XAG	8	0.51%	XF	4	0.26%
XAH	1	0.06%	XG	1	0.06%
XAJ	52	3.32%	XH	4	0.26%
XAK	21	1.34%	XL	11	0.70%
XAM	59	3.77%	XM	10	0.64%
XAN	5	0.32%	XN	16	1.02%
XAP	41	2.62%	XP	7	0.45%
XAT	2	0.13%	XQ	14	0.89%
XAU	4	0.26%	XS	24	1.53%
XB	19	1.21%	XW	36	2.30%
XBB	67	4.28%	XY	63	4.03%
XBB.1	88	5.62%	XZ	59	3.77%
XBB.2	206	13.16%	**TOTAL**	**1,565**	**100%**

The Variants of Concern (VOCs) began to emerge around November 2020, with the Alpha variant being the most prominent at that time [38]. For classification purposes, we refer to variants identified between January 2020 and November 2020 as pre-VOC variants. Table 1 shows the compilation of non-recombinant sequences by variants (pre-VOC and VOC).

The prevalent variants are, first and foremost, the collection known as pre-VOC, with 218,198 sequences, followed by Alpha, with 198,722 sequences, Delta, with 325,285 sequences, and, above all, Omicron, along with all its sub-variants, totaling 748,635 variant sequences. The impact of the remaining variants has been more limited, due to the dominance of those prevalent ones that gained an evolutionary advantage. Therefore, the rest of the variants represent only 48,888 sequences.

Table 2 shows the SARS-CoV-2 compilation of recombinant sequences by variants. The compilation of recombinant sequences is more balanced than in the case of non-recombinants, with a slight prevalence of XBB sub-variants over the others.

### 2.2 Dataset design

The prevalent variants throughout the SARS-CoV-2 pandemic (and its worst moments) were non-recombinants [42]. Primarily for this reason, the number of non-recombinant variant sequences is substantially greater than that of recombinants. In response to this disparity, we opted to implement a subsampling technique in the larger non-recombinant dataset. This strategy involves selecting a random, representative subsample from the larger category, thereby equalizing the number of data points between both categories. This, in turn, helps mitigate potential biases in our analysis and enhances the validity of our results [43]. We randomly selected 1,565 non-recombinant sequences to work with a balanced dataset. To ensure the generalization of our results, we performed a significant and sufficient number of different subsamplings. In this case, 10 subsamplings, labeled with sequential numbers from 01 to 10 (SUB_01—SUB_10).

Once the subsampling of non-recombinant sequences was completed, they were randomly distributed among the Training, Validation, and Test sets [44], as detailed in Table 3, which illustrates the structure of each dataset generated by subsampling.

**Table 3:** Dataset structure. We conducted all experiments using three balanced datasets between both categories, allocating 60% to the Training Set, 20% to the Validation Set, and 20% to the Test Set.

Dataset	Non-recombinant	Recombinant	TOTAL
Training	939	939	1,878 (60%)
Validation	313	313	626 (20%)
Test	313	313	626 (20%)
**TOTAL**	**1,565**	**1,565**	**3,130**

### 2.3 Generation of genomic spectrograms

The generation of spectrograms follows the procedure corresponding to the superposed spectrograms in our previous work [45], were we applied transfer learning to a pre-trained Convolutional Neural Network (CNN) VGG-16 [46] using the ImageNet dataset [47]. This network was trained to detect the recombinant feature in complete HIV-1 sequences.

In this spectrogram representation, the z-axis represents the summation of values from each of the four nucleotide types along the z-axis.

$$\begin{array}{c} S=S_{a}+S_{g}+S_{c}+S_{t} \end{array}$$ (1)

**Fig 1:**
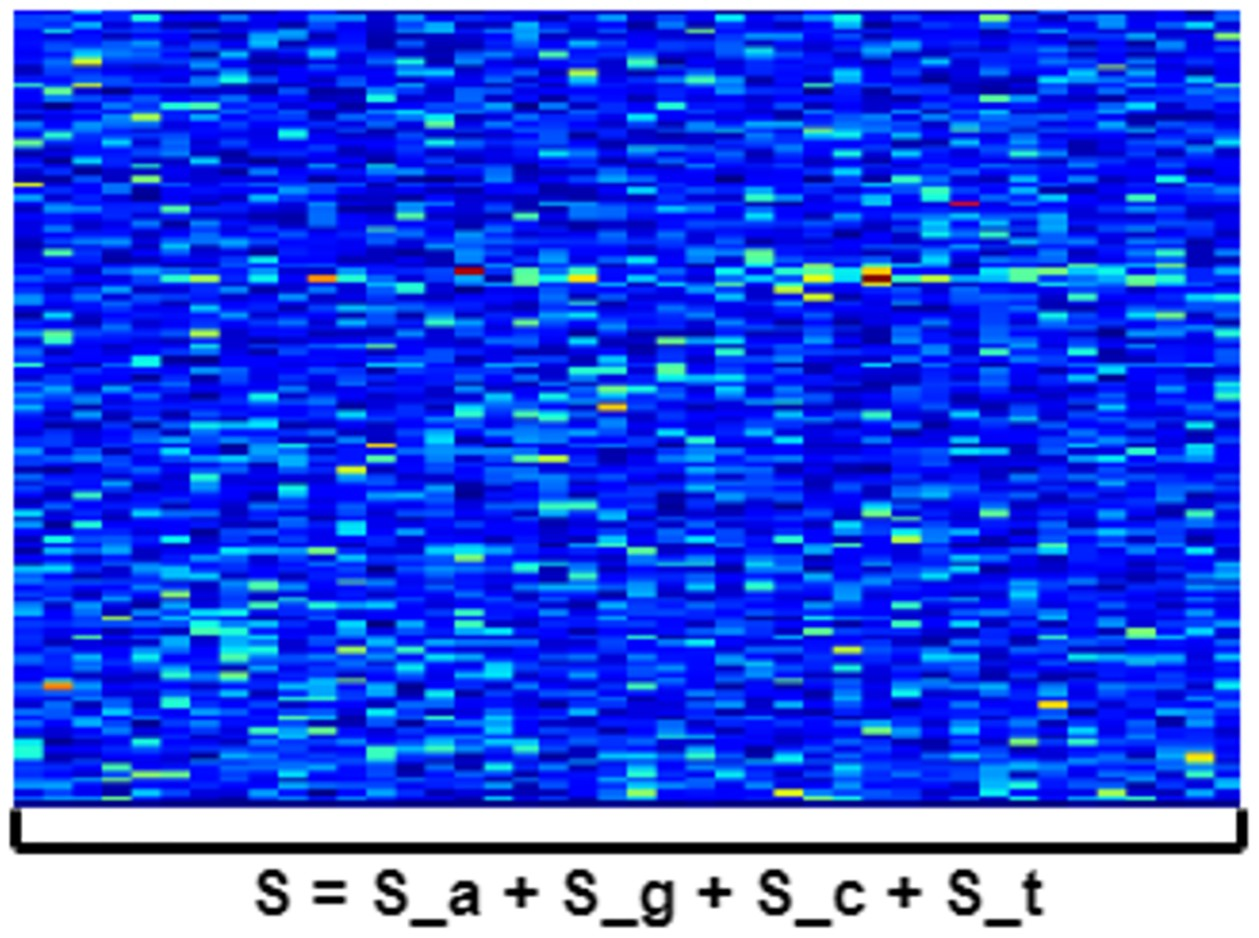
HIV-1 genomic spectrogram scheme. In the case of HIV-1, the length of the x-axis is approximately 10,000 nucleotides.

**Fig 2:**
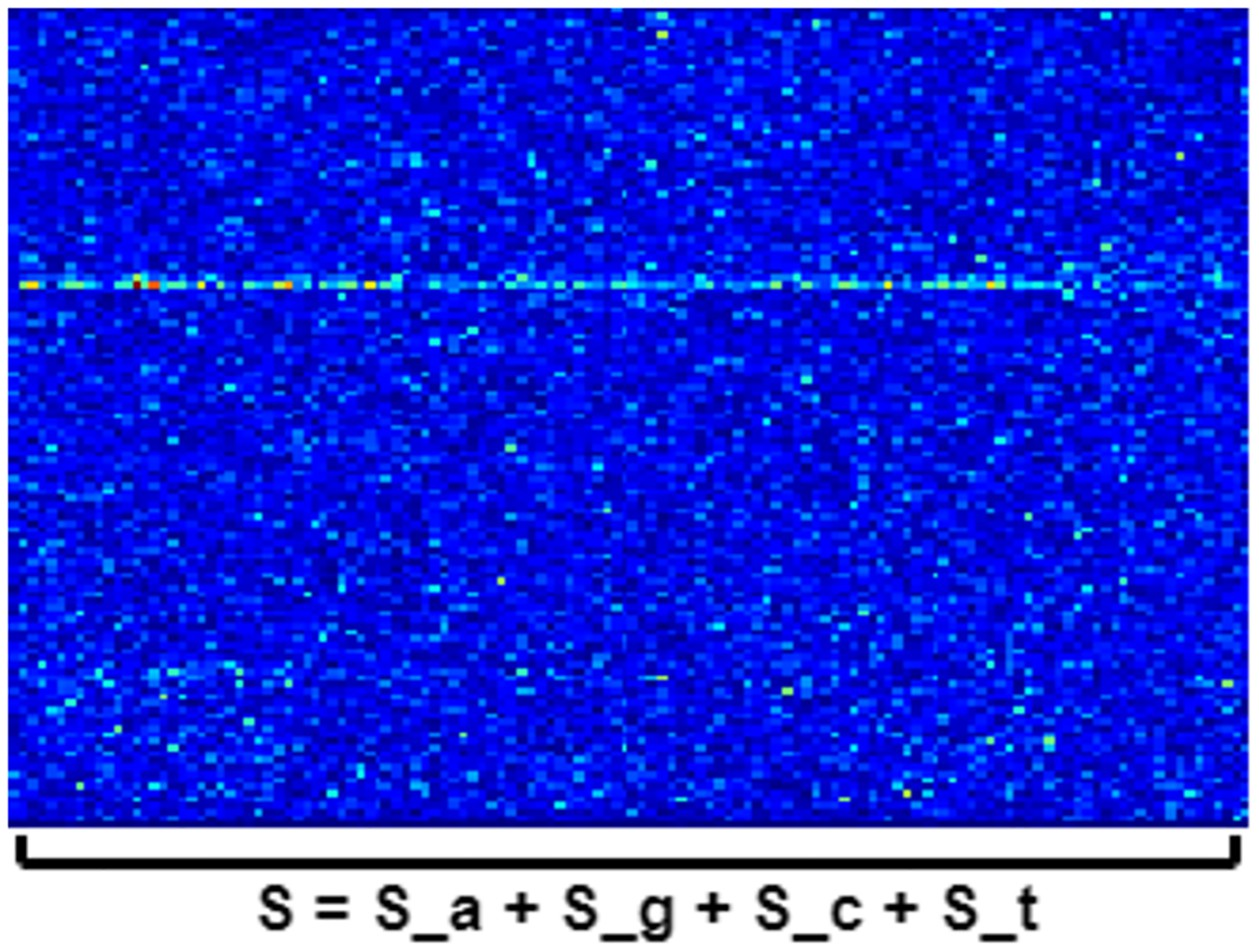
SARS-CoV-2 genomic spectrogram scheme. In the case of SARS-CoV-2, the length of x-axis is around 30,000 nucleotides.

Fig 1 shows an example of the genomic spectrogram of HIV-1, and Fig 2 shows that of SARS-CoV-2. The length of x-axis matches the genome length.

In both cases, the y-axis represents a frequency range from 0 to 0.5 Hz. The z-axis represents the spectrogram computation with an applied jet colormap scale. Lower values are represented in blue with a progressive scaling towards the color red, which represents higher values, transitioning through intermediate colors in the range of greens, yellows, and oranges.

We generated spectrograms of both datasets using Python, the Scipy library, scipy.signal.spectrogram. The length of the SARS-CoV-2 genome is approximately three times greater than that of the HIV-1 genome, and this contrast is evident in the spectrogram due to the fixed value of 256 used as the length of each time segment for FFT calculation (nperseg). In the spectrogram of SARS-CoV-2, the x-axis is three times longer than in the case of HIV-1, resulting in smaller color points along the z-axis [48].

In both cases, the horizontal line at *f* = 1/3 is clearly visible. This observation may be related to the conversion of three nucleotides into a single amino acid in the coding regions of the genome [49]. It is expected that this line will be sharp in these coding regions but less pronounced in non-coding regions. In the case of viruses, a significant portion of the genome is coding [50]. Hence, this line is perfectly visible throughout nearly the entire genomic spectrogram. The appearance of this line at *f* = 1/3 is an indicative of a correct generation of genomic spectrograms.

This graphical representation of genome in the frequency spectrum allows for a more accurate identification of the genome regions crucial for the recombinant feature.

### 2.4 Two-stage transfer learning

**Fig 3:**
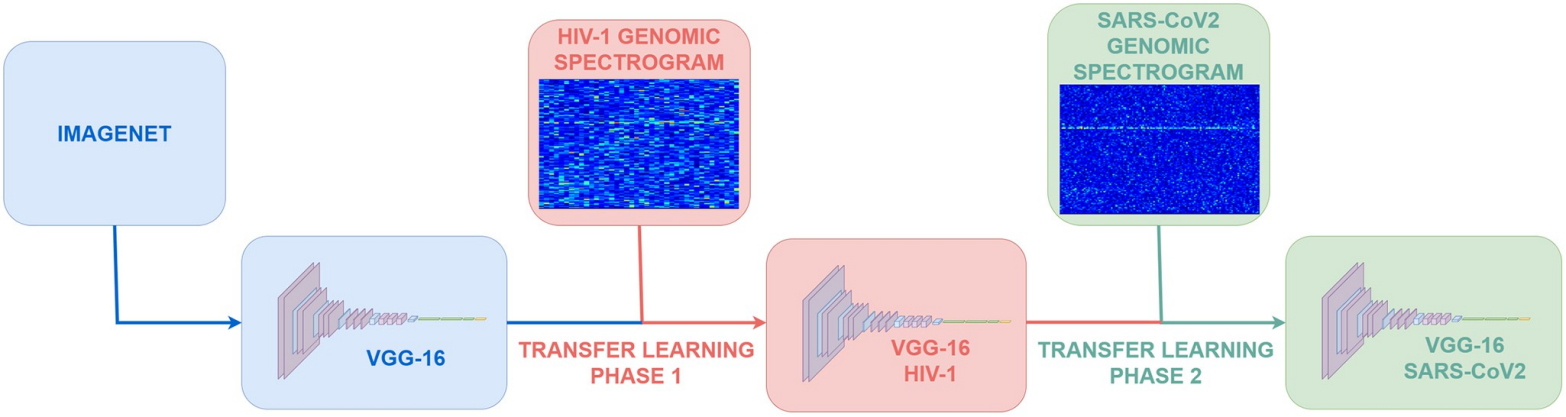
Two-stage transfer learning methodology. We started with a pre-trained VGG-16 using the ImageNET dataset. In Phase 1, we applied transfer learning to the genomic spectrogram dataset of complete HIV-1 sequences to detect the recombinant feature. In Phase 2, we applied transfer learning once again to the resulting network from Step 1 (VGG-16 HIV-1) using a genomic spectrogram dataset of complete SARS-CoV-2 sequences to also detect the recombinant feature (VGG-16 SARS-CoV-2).

We performed a two-stage transfer learning process as indicated in Fig 3. The first of these stages started from the network derived from [45] for superposed spectrograms.

All the experiments were performed using the MATLAB2021b App Deep Learning Designer.

### 2.5 Test bench

We conducted a Test Bench on each of the 10 subsamplings, evaluating hyperparameter values with a fixed Learning Rate of 0.0001, a fixed Batchsize of 52, and varying the number of Epochs at 10, 25, 50, 75, 100, 150, 200, and 250.

Consequently, we conducted a total of 80 experiments, resulting in 80 VGG-16 trained through Two-Stage Transfer Learning.

We conducted preliminary tests with a Batchsize of 128 and a Learning Rate of 0.01, which yielded suboptimal results. Consequently, we found that the optimal values for Batchsize and Learning Rate in this second stage of transfer learning align with those used in the first stage (HIV-1).

### 2.6 Performance measurement

As performance metrics, alongside Validation Accuracy and Test Accuracy, we calculated the Area Under the Curve (AUC) and the Confusion Matrix on the Test Set for all experiments.

Our criterion for determining the optimal configurations was based on selecting those that, with remarkable values of AUC and Validation Accuracy, not only achieve the highest Test Accuracy but also maintain a balance in both categories (recombinant and non-recombinant) [51].

We calculated all performance measurements using MATLAB2021b.

### 2.7 Interpretability analysis

We applied interpretability techniques to discern, via heatmaps, the critical influences on the outputs of each of the generated models. Our tool of choice for pinpointing the regions of the genome where the network looks to make decisions is Grad-CAM [52]. It offers greater visual clarity compared to other tools like LIME [53] or Gradient Attribution [54], albeit at the cost of some precision.

**Fig 4:**
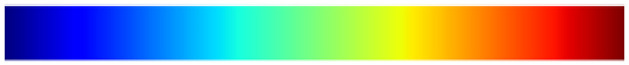
Jetmap color scale. Color values increase from blue to red, with blue indicating lower values and red indicating higher values, transitioning through intermediate colors such as greens, yellows, and oranges.

The color scale applied to these heatmaps is a jet map, whose color distribution based on the value of the scoremap at each point is shown in Fig 4.

**Fig 5:**
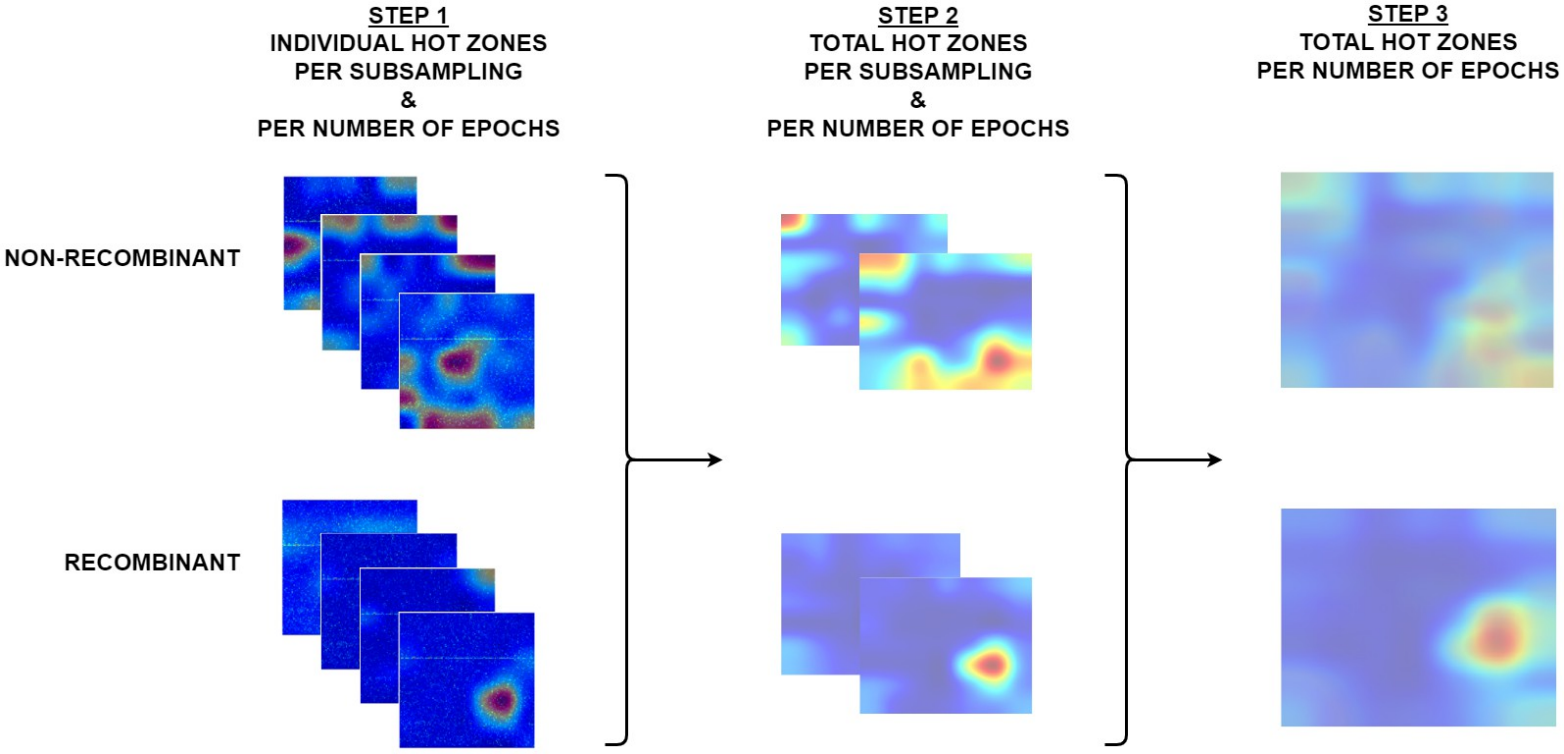
Three-step interpretability. Considering that we conducted 80 experiments (test bench applied to 10 subsamplings), the first step involved a total of 50,080 images, taking into account that each complete test set contains 626 sequences. The second step involves 160 images (80 experiments and 2 categories). And the third step involves a total of 16 images per category and number of Epochs.

To process the Grad-CAM results, we performed a three-step image processing to progressively determine the relevant Total Hot Zones in the recombinant feature. Fig 5 graphically illustrates the interpretability analysis methodology.

In the first step, we obtained the scoremaps for each sequence, in each subsampling, and for each hyperparameter configuration. In the second step, we calculated the total hot zones per category in each subsampling and for each hyperparameter configuration. In the third step, we calculated the total scoremap image per category for each hyperparameter configuration, considering the ten subsamplings. The result of this third step represents the relevant hot zones across the set of subsamplings by category.

For the calculation of the images resulting from Steps Two and Three, we applied two different techniques, which were determined by their input data. For the calculation of the Total Hot Zones in Step Two, we processed the scoremaps (numerical matrices corresponding to each of the sequences) and summed the numerical values at each position in the matrix.

$$\begin{array}{c} H\left(STEP2\right)=\sum_{i=1}^{n}H{\left(STEP1\right)}_{i} \end{array}$$ (2)

The scalar summation of Grad-CAM (Class Activation Maps) and the generation of an average heatmap are two different approaches to summarize and visualize the importance of regions of interest in the images. Each approach has specific advantages.

The arithmetic summation (Step Two) allowed, in a straightforward and computationally efficient manner, to calculate the total hot zone for each of the 80 experiments conducted, without any inherent loss of image processing accuracy. Subsequently applying the same color map to this resulting numerical matrix allowed us to obtain the total hot zone image per category for each subsampling and each experiment in the test bench.

**Fig 6:**
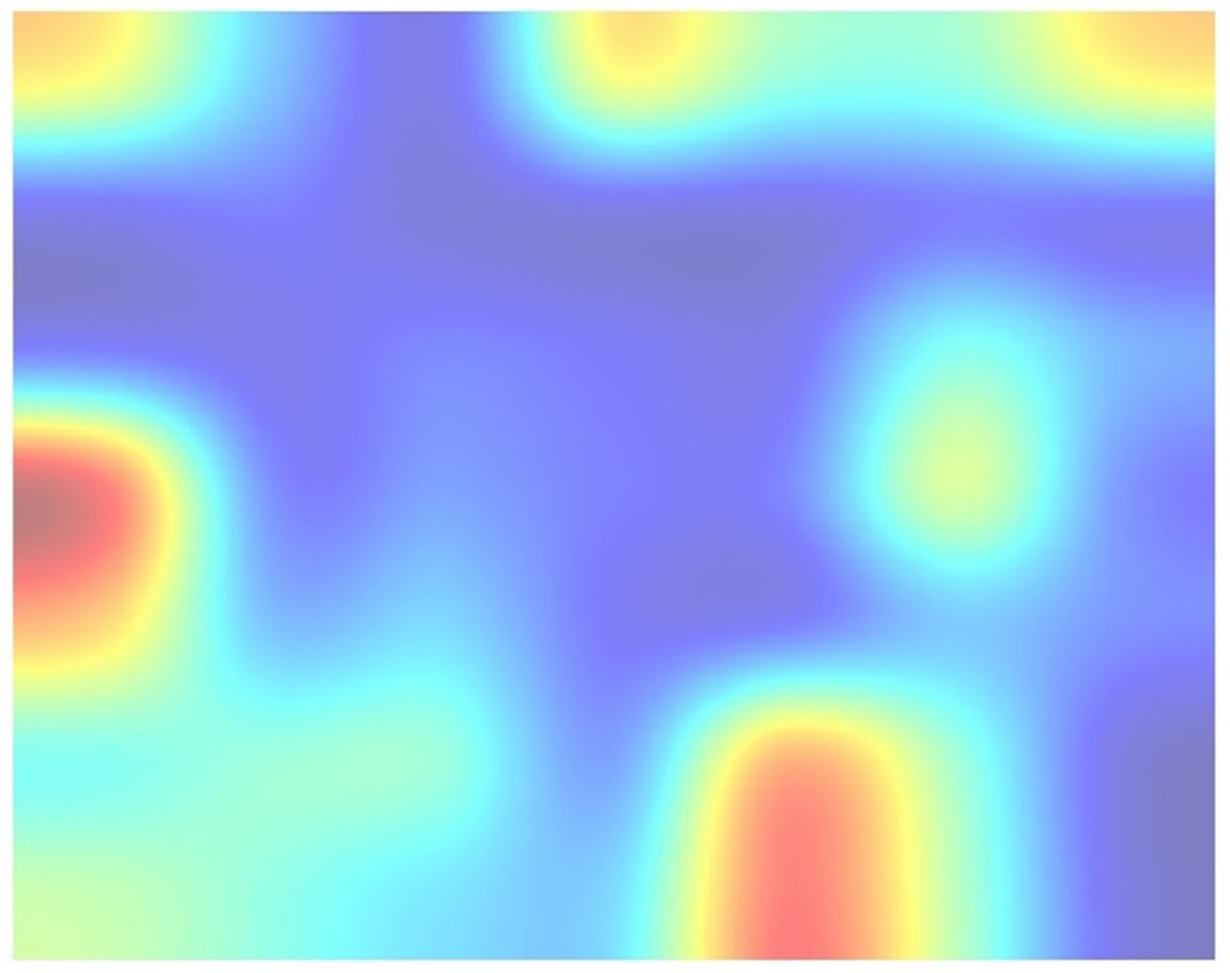
Total hot zones (SUB_05, 200 Epochs) in non-recombinants. The non-recombinant hot zones are more variable and diffuse.

**Fig 7:**
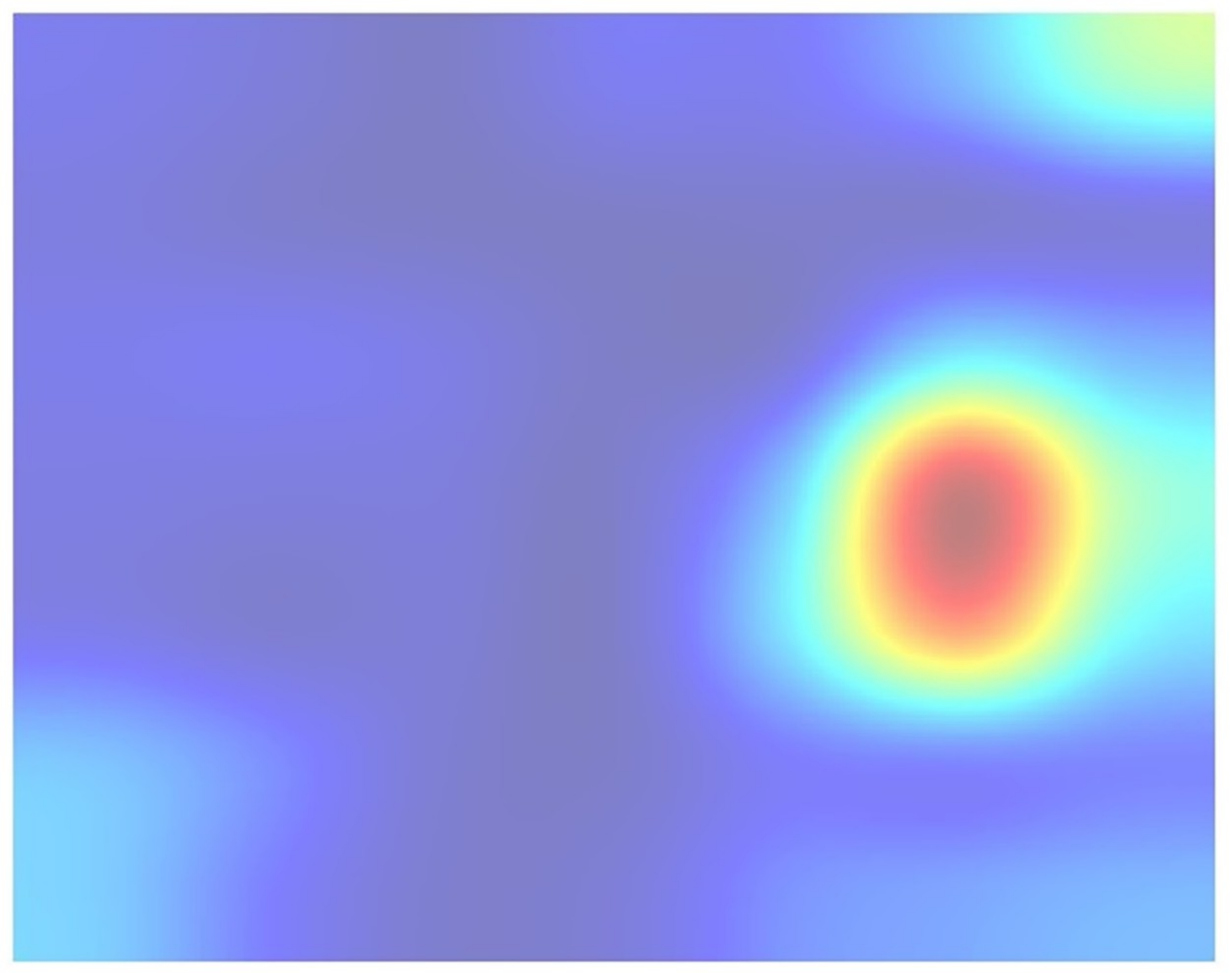
Total hot zones (SUB_05, 200 Epochs) in recombinants. However, in the case of recombinant sequences, a clear, well-defined hot zone appears, concentrated in the same area.

This way, we obtained a clear and representative heatmap of the hot zones in the dataset. For example, the total hot zones per category in subsampling 05 for 200 Epochs are shown in Figs 6 and 7. Fig 6 shows the total hot zones in the case of non-recombinants and Fig 7 in the case of recombinants.

In Step Three, we generated composite hot zone images for each hyperparameter configuration across the 10 subsamplings. These images represent the arithmetic mean of the hot zones obtained from each subsampling.

$$\begin{array}{c} H\left(STEP3\right)=\frac{1}{n}\sum_{i=1}^{n}H{\left(STEP2\right)}_{i} \end{array}$$ (3)

Where n represents the number of subsamplings, in this case, 10.

Step Three visually represents the common and relevant hot zones for the recombinant feature considering the 10 subsamplings. Since we only had 10 images per category (non-recombinant and recombinant) in the initial data, we implemented image processing techniques to calculate a weighted average of each pixel in the image set, creating a comprehensive total image of the hot zones across the 10 subsamplings. This technique allowed us to diminish the significance of noisy or atypical regions in the individual maps, achieving a generalized view of the important areas in each category.

The application of both image processing techniques enabled us to attain a more comprehensive view of the total hot zones throughout the whole process.

After obtaining the total hot zone in Step Three, we modified the color scale to visually enhance the hot zones. We achieved this by normalizing the average matrix so that the lowest value equals 0, and the highest equals 255 as follows [55]:

$$\begin{array}{c} \text{Norm.}\;\text{Avg.}\;\text{Matrix}=(\frac{\text{Avg.}\;\text{Matrix}-\text{Avg.}\;\text{Matrix}\;\text{Min.}}{\text{Avg.}\;\text{Matrix}\;\text{Max.}-\text{Avg.}\;\text{Matrix}\;\text{Min.}})\times 255 \end{array}$$ (4)

Where Avg. Matrix represents the resulting average matrix from the Step Three. Avg. Matrix Min. represents the minimum value contained in Avg. Matrix, and Avg. Matrix Max. represents the maximum value.

Finally, in one last adjustment, we generated the negative of the resulting matrix so that the minimum value appears as a light color and the maximum as a dark color. All of this was done with the aim of enhancing visualization and highlighting the location of the total hot zones per epochs [56].

All of these processes were conducted using MATLAB 2021b along with Python library functions, utilizing cv2 for image processing and numpy for multidimensional array manipulation and algebraic operations [57].

## 3 Results and discussion

### 3.1 Results per subsampling

S1 Appendix includes the complete set of results per subsampling in terms of performance. The performance metrics are detailed in Section 2.6. Those configurations (specified by the number of epochs) that yielded best results, meaning highest test accuracy values and a more balanced distribution of hit rates between the two categories, are highlighted in green.

We evaluated the balance between the two categories by computing the Standard Deviation (SD) between the test accuracy values for recombinants and non-recombinants.

Therefore, we considered the optimal configurations to be those with the highest hit rate in the test set and the most balanced distribution (lower SD between the test accuracies of both categories).

**Fig 8:**
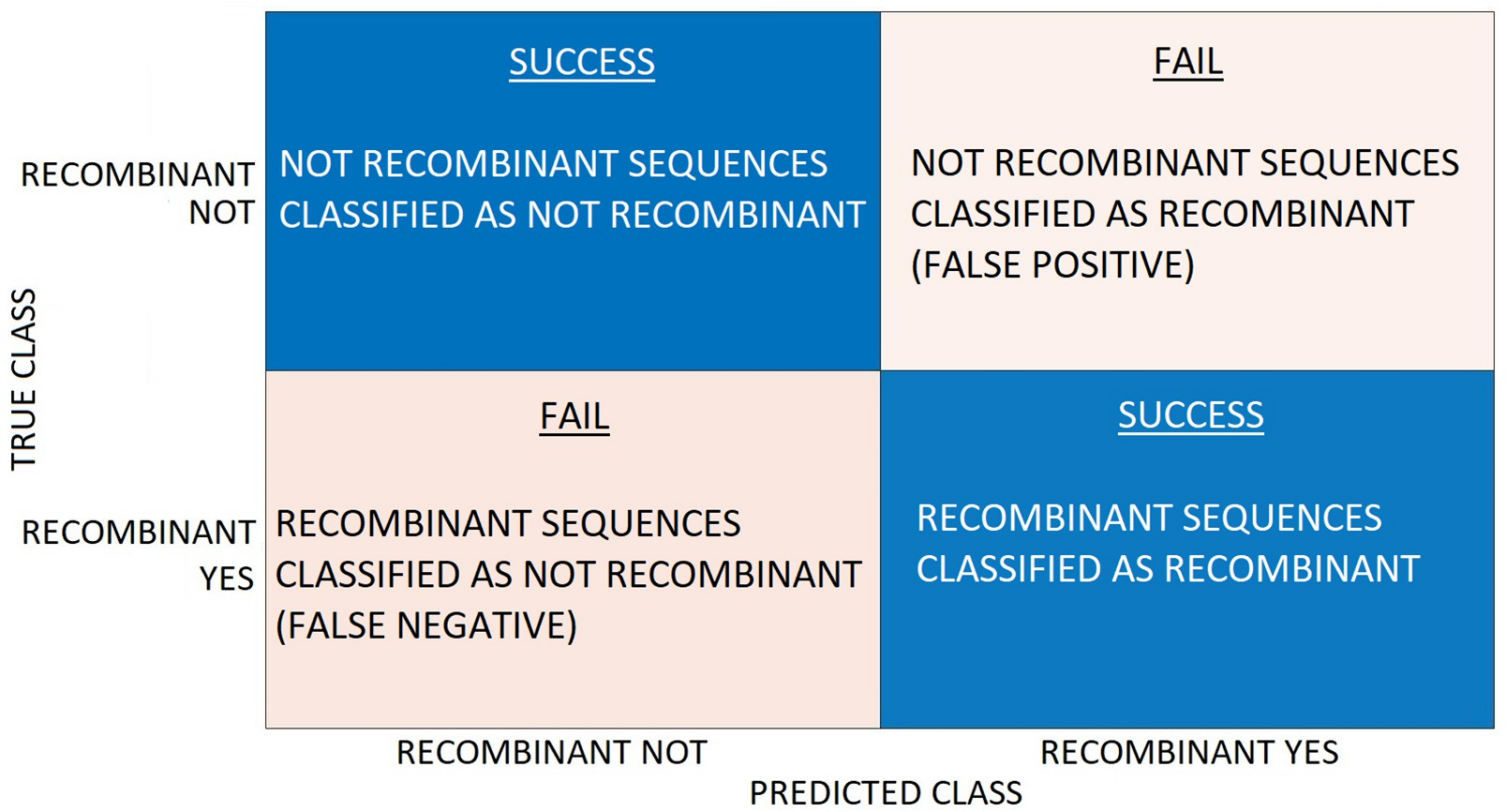
Confusion matrix scheme. The top row corresponds to the Non-recombinant Category, and the bottom row to the Recombinant Category.

Fig 8 graphically represents the confusion matrix scheme outlined in Table 4.

**Table 4:** Best results per subsampling. The best configurations feature 200 Epochs in 80% of the subsamplings.

**SUBSAMPLING 01**				
**Epochs**	**AUC**	**Validation Accuracy**	**Test Accuracy**	**Confusion Matrix**
150	0.9818	94.25%	96.01%	302–11 14–299
200	0.9823	94.57%	96.01%	302–11 14–299
**SUBSAMPLING 02**				
**Epochs**	**AUC**	**Validation Accuracy**	**Test Accuracy**	**Confusion Matrix**
150	0.9806	94.57%	95.85%	303–10 16–297
250	0.9796	95.05%	95.53%	298–15 13–300
**SUBSAMPLING 03**				
**Epochs**	**AUC**	**Validation Accuracy**	**Test Accuracy**	**Confusion Matrix**
200	0.9824	95.21%	94.73%	297–16 17–296
**SUBSAMPLING 04**				
**Epochs**	**AUC**	**Validation Accuracy**	**Test Accuracy**	**Confusion Matrix**
200	0.9808	96.17%	95.53%	304–9 19–294
**SUBSAMPLING 05**				
**Epochs**	**AUC**	**Validation Accuracy**	**Test Accuracy**	**Confusion Matrix**
200	0.9698	95.53%	93.13%	294–19 24–289
250	0.9732	95.21%	93.13%	288–25 18–295
**SUBSAMPLING 06**				
**Epochs**	**AUC**	**Validation Accuracy**	**Test Accuracy**	**Confusion Matrix**
200	0.9782	94.57%	94.57%	297–16 18–295
**SUBSAMPLING 07**				
**Epochs**	**AUC**	**Validation Accuracy**	**Test Accuracy**	**Confusion Matrix**
150	0.9824	94.73%	95.37%	309–4 25–288
200	0.9806	94.73%	95.21%	305–8 22–291
**SUBSAMPLING 08**				
**Epochs**	**AUC**	**Validation Accuracy**	**Test Accuracy**	**Confusion Matrix**
150	0.9769	91.05%	91.69%	275–38 14–299
200	0.9754	91.69%	91.69%	275–38 14–299
**SUBSAMPLING 09**				
**Epochs**	**AUC**	**Validation Accuracy**	**Test Accuracy**	**Confusion Matrix**
100	0.9842	94.89%	96.17%	305–8 16–297
**SUBSAMPLING 10**				
**Epochs**	**AUC**	**Validation Accuracy**	**Test Accuracy**	**Confusion Matrix**
200	0.9728	93.45%	95.05%	302–11 20–293
250	0.9729	93.77%	95.05%	302–11 20–293

Table 4 summarizes the best configurations for each of the 10 generated subsamplings. These are the ones that exhibit the highest test accuracy values with a greater balance between both categories.

### 3.2 Results per number of epochs

Complete results are provided in S2 Appendix Table 5 summarizes the most relevant data.

**Table 5:** Best results per number of epochs. We include the mean value and standard deviation of AUC, the mean Test Accuracy for non-recombinant and recombinant sequences, and the standard deviation between the mean test accuracy values of recombinants and non-recombinants. As a measure of the network’s accuracy, the total number of sequences in each category within the test set is 313.

	AUC		Non-rec. Test Acc.	Rec. Test Acc.	Inter Category
EPOCHS	Avg.	SD	Avg.	Avg.	SD
10	0.9013	0.0201	249.2	263.8	10.32
25	0.9535	0.0112	289.6	253.9	25.24
50	0.9696	0.0096	291.7	285.2	4.60
75	0.9729	0.0053	295.8	286.6	6.51
100	0.9761	0.0070	295.5	290.1	3.82
150	0.9787	0.0043	300.0	292.2	5.52
200	0.9785	0.0046	297.6	294.6	2.12
250	0.9785	0.0038	298.6	292.5	4.31

The configurations corresponding to 10 and 25 Epochs yielded deficient results in terms of test accuracy in both categories, and the results are unbalanced, making them inappropriate configurations due to the insufficient training with such low values of the number of epochs. The qualitative advantage of 200 Epochs over 150 or 250 is its higher degree of balance between the test accuracy of both categories. Therefore, although these three configurations exhibit high hit rates on the test set, in the case of 200 Epochs, the minimum value of Inter-Category SD was achieved.

**Fig 9:**
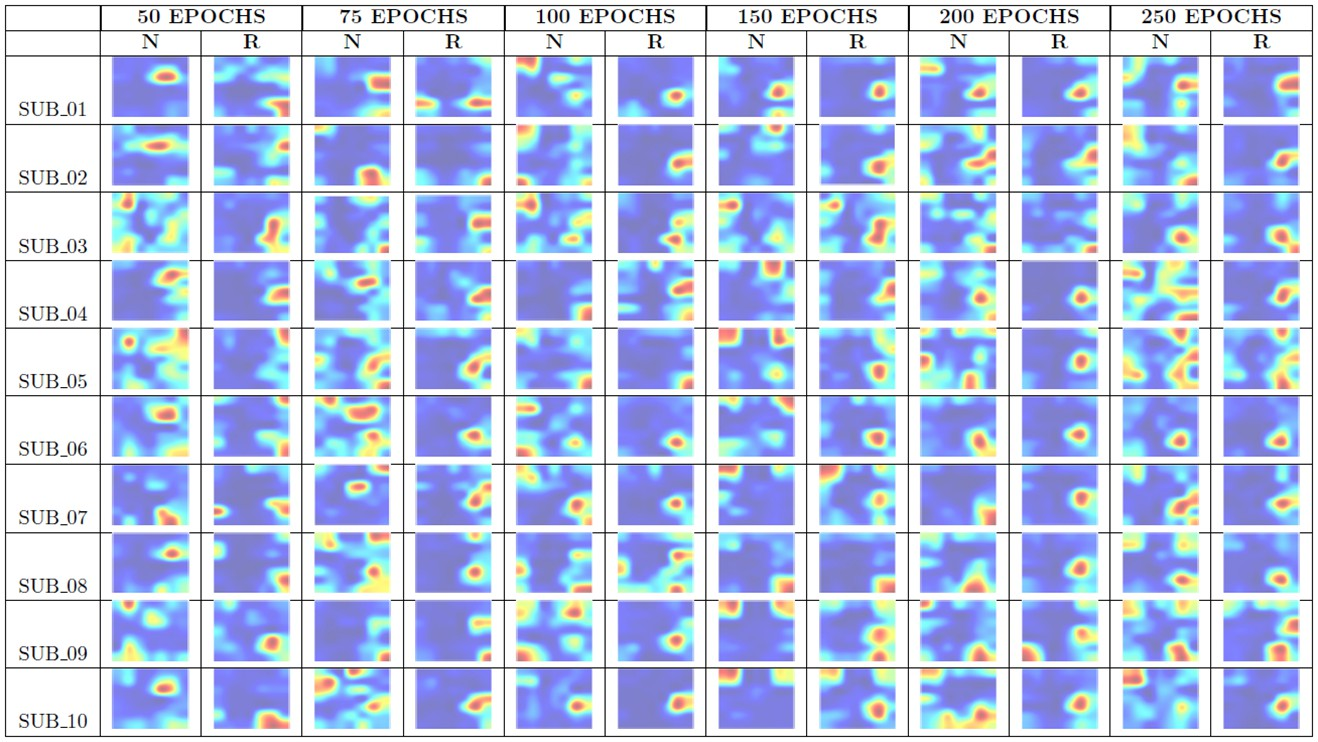
Summary table of total hot zones Step Two. N stands for the non-recombinant category, and R stands for the recombinant one.

Fig 9 displays the summary of the total hot zones per category for each subsampling by number of epochs (Step Two). From the images shown in Fig 9, we generated the corresponding images for Step Three, that is, the weighted average hot zones for each configuration.

We omitted 10 and 25 Epochs as their performance ratios were not suitable, possibly due to insufficient training.

**Fig 10:**
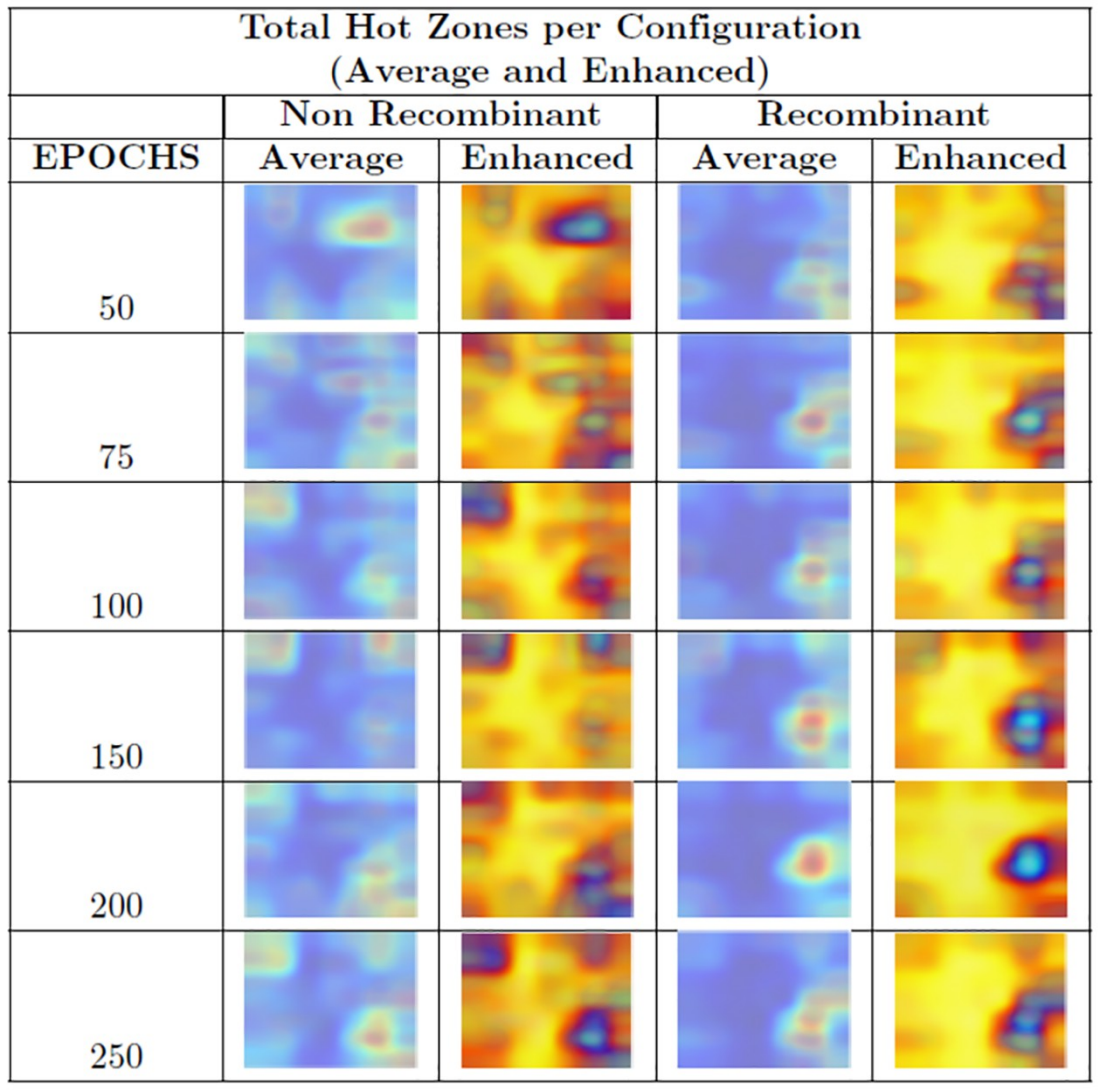
Total hot zones per configuration. The average hot zones represent the hot zones for each number of epochs across the 10 subsamplings. The enhanced figures are the average hot zones with color scale modifications to clarify the relevant hot zones in each category.

Fig 10 displays the weighted average hot zones for each configuration (Step Three) and their enhanced counterparts. In the case of non-recombinants, the main hot zones are more diffuse, as opposed to the greater sharpness observed in recombinants. For the latter, all configurations clearly converge towards a single area.

We processed 17,215 complete sequences of SARS-CoV-2, utilizing virtually all available complete recombinant sequences at the beginning of the experimentation. We are aware that handling 10 subsamplings of the total non-recombinant pool involved processing only approximately 1% of the available non-recombinant sequences. Nevertheless, the results obtained are significant, especially in the recombinant category, indicating that it is a representative sample. Our results confirm this point.

### 3.3 Optimal configuration selection

The configurations with the highest number of correct predictions in the test set are 150 epochs and 200 epochs, with a total test accuracy in both cases of 94.60%. At similar test accuracy values, maintaining the test accuracy constant, improving the balance between categories leads to superior model performance.

**Fig 11:**
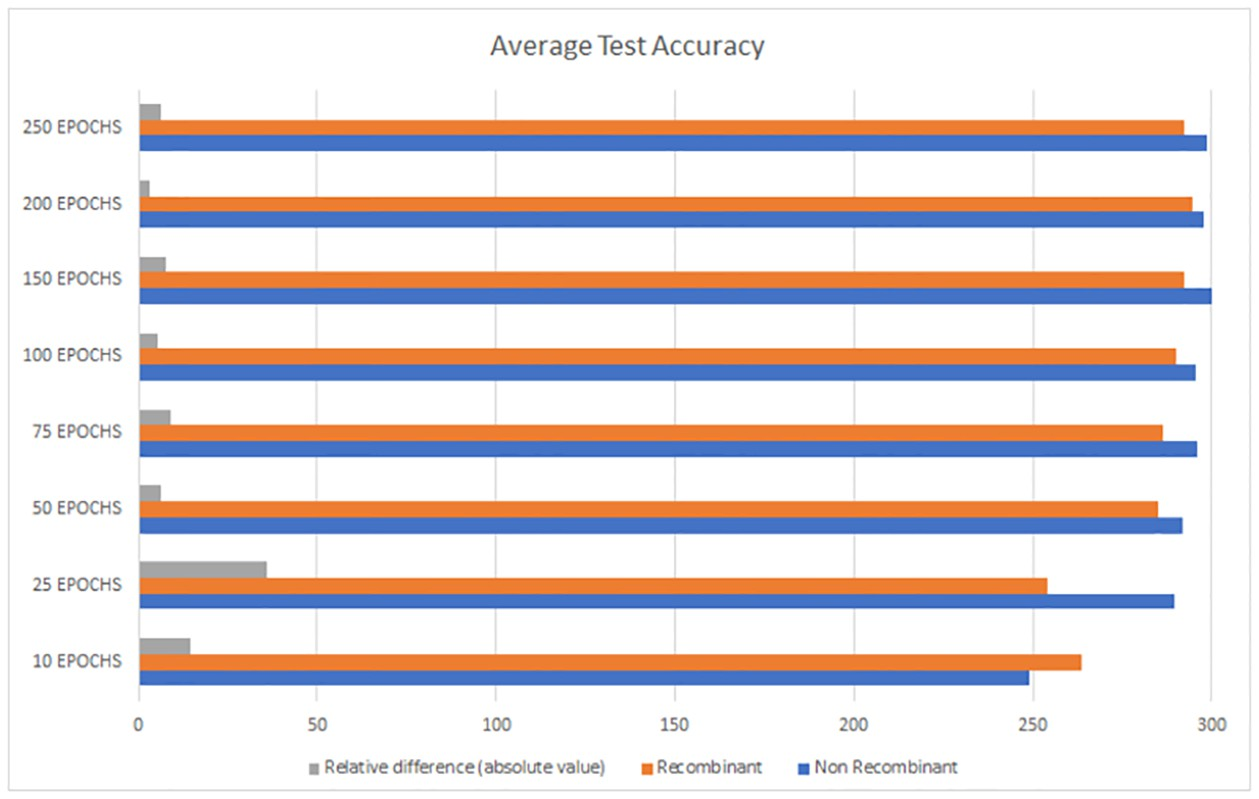
Average test accuracy for recombinants (in orange) and non-recombinants (in blue). In gray, we depicted the relative difference between these mean absolute values.

**Fig 12:**
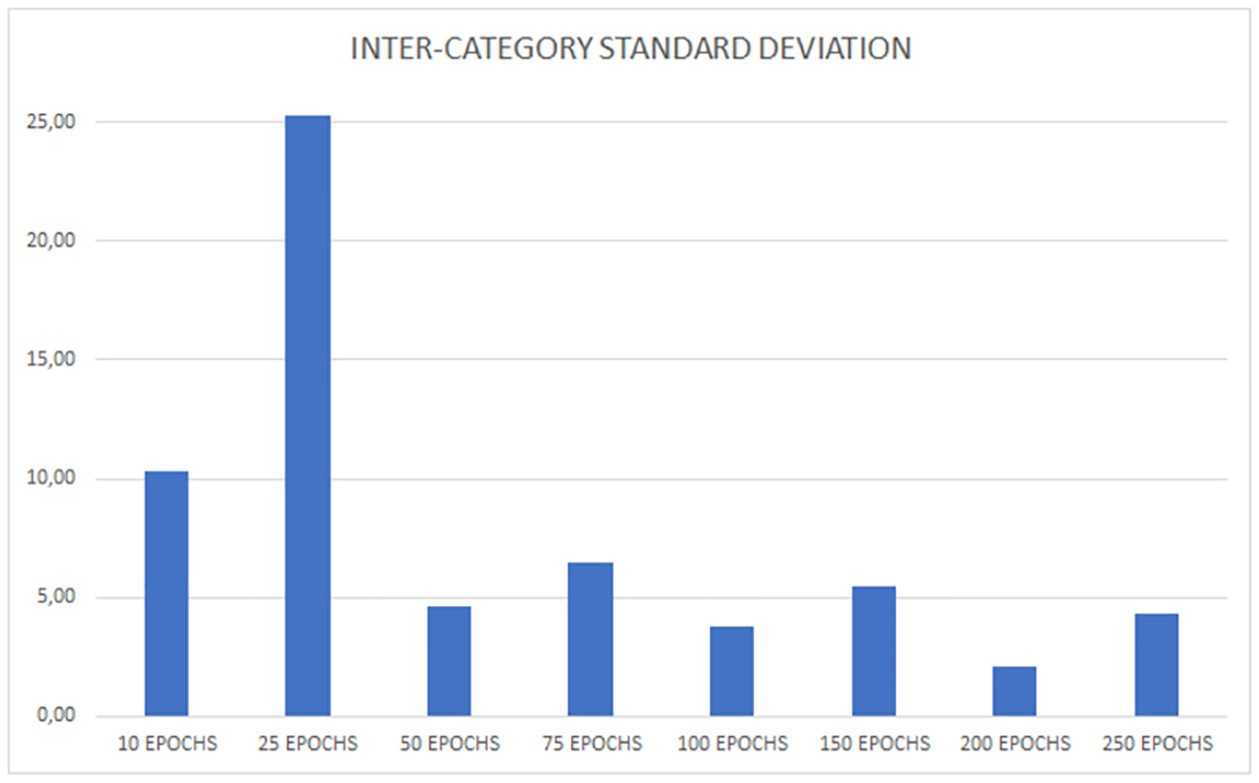
SD values between the average test accuracy values for recombinants and non-recombinants.

As we discussed in previous sections, the decision criteria cannot be based solely on the mere measure of total accuracy in a single category or in both categories combined. We require balanced results, hence the need to include inter-category SD in the decision criteria. In this case, the 200 Epochs configuration achieves remarkable accuracy rates in both categories (see Fig 11), and the relative difference in absolute terms is minimal. Indeed, the inter-category SD is the lowest (see Fig 12).

### 3.4 Reference sequence

To identify the genomic regions where the hot zones are located, we relied on the Severe Acute Respiratory Syndrome Coronavirus 2 isolate Wuhan-Hu-1, complete genome NCBI Reference Sequence: NC_045512.2 [58]. The location of each structural, non-structural, and accessory protein is indicated in Table 6.

**Table 6:** Location of protein coding regions in the SARS-CoV2 Wuhan-Hu-1 reference genome sequence (NC_045512.2) [58]. The “Beginning” column specifies the first nucleotide of the corresponding protein, while the “End” column indicates the last nucleotide.

Protein coding region in NC_045512.2 genome sequence		
	Beginning	End
**5’UTR**	1	265
**ORF1ab**	266	21555
**S**	21563	25384
**ORF3a**	25393	26220
**E**	26245	26472
**M**	26523	27191
**ORF6**	27202	27387
**ORF7a**	27394	27759
**ORF7b**	27756	27887
**ORF8**	27894	28259
**N**	28274	29533
**ORF10**	29558	29674
**3’UTR**	29675	29903

Based on the consensus reference sequence, we constructed a scaled graphical representation of the SARS-CoV-2 genome, which will serve as a pivotal tool for the precise identification of regions involved in the recombinant feature.

### 3.5 Analysis of non-recombinant results

Once it is established that the optimal configuration corresponds to 200 Epochs, the next step is to identify the high-impact hot zones for classifying a sequence as non-recombinant. To do this, we calculate the overall average image (Step 3), the enhanced image, and the localization of the epicenters of the main hot zones on the x-axis (indicating genomic region involvement) and the y-axis (frequency range identification), all in accordance with the guidelines outlined in Section 2.7.

**Fig 13:**
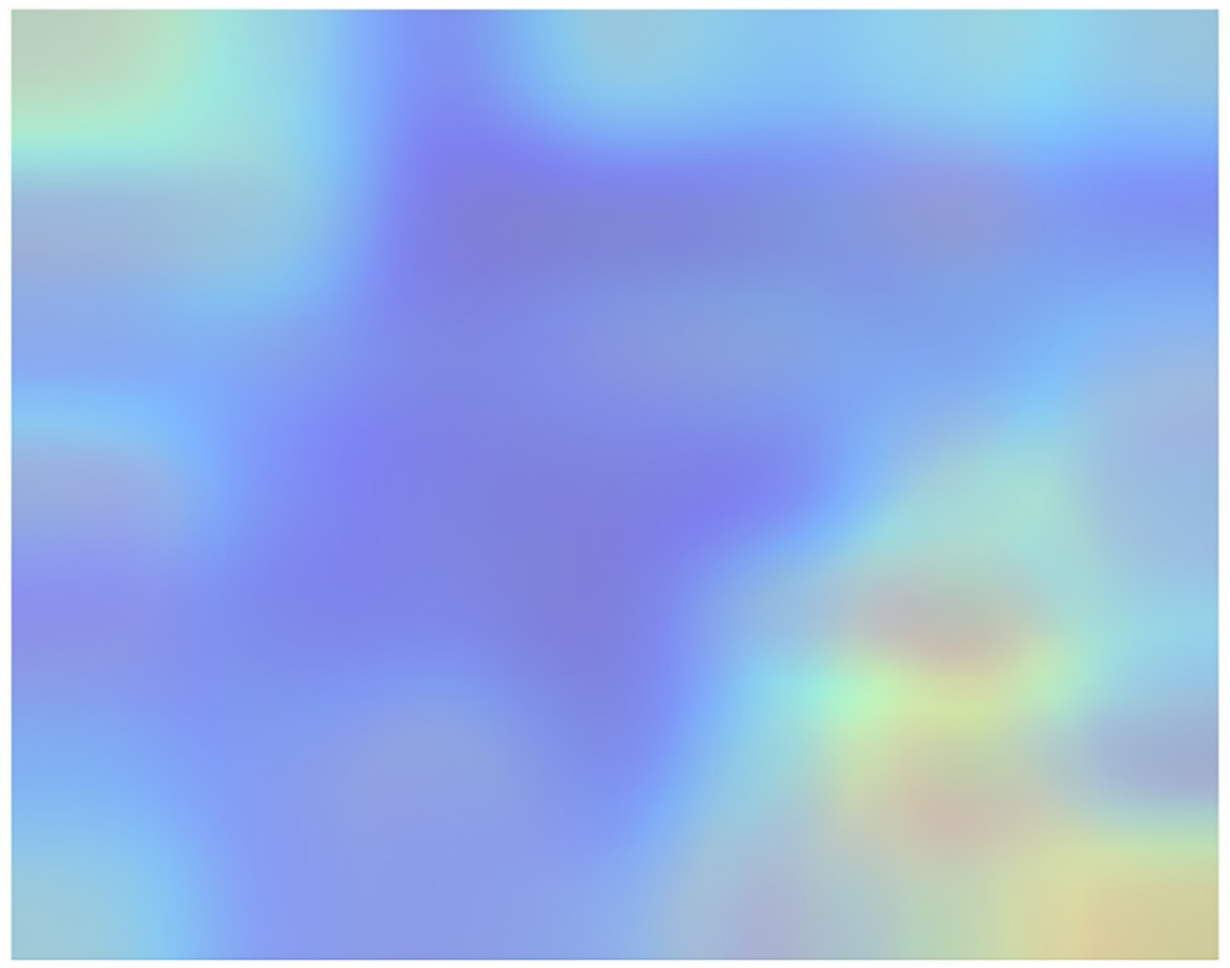
Non-recombinant total hot zones resulting from the 10 subsamplings for 200 Epochs.

**Fig 14:**
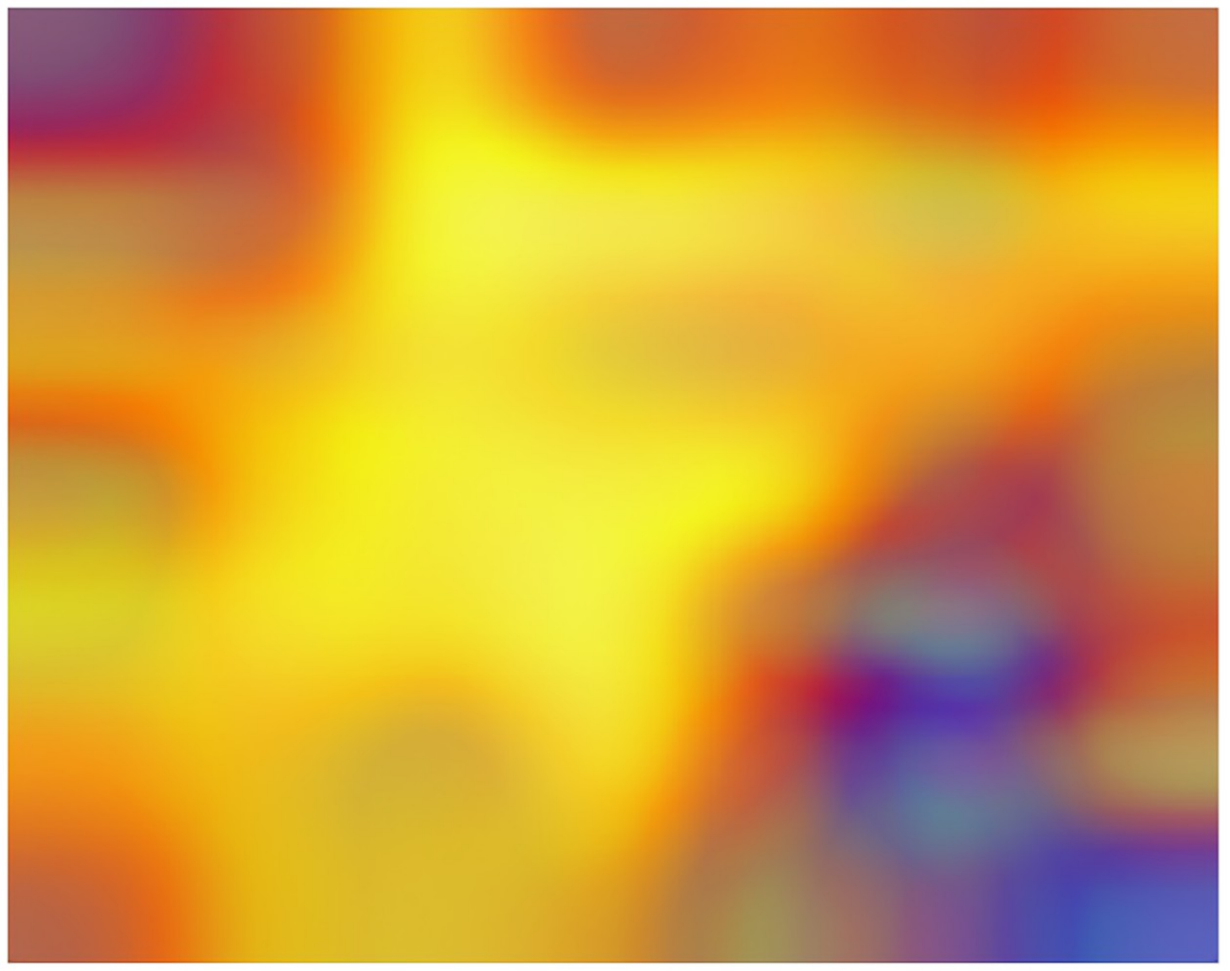
Image resulting from color scale enhancement for better non-recombinant hot zone identification.

**Fig 15:**
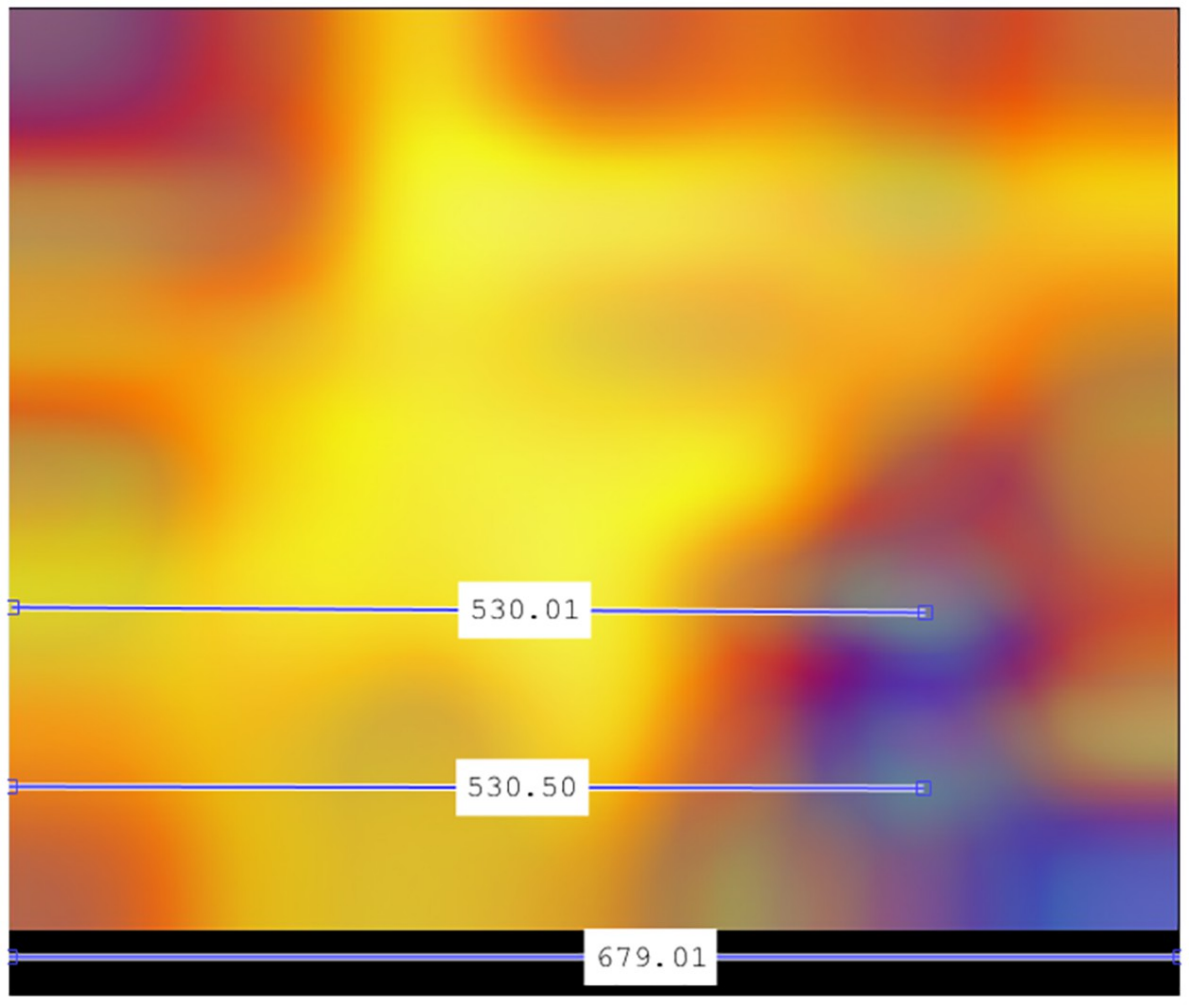
Horizontal positioning of the epicenters of the main non-recombinant hot zones.

**Fig 16:**
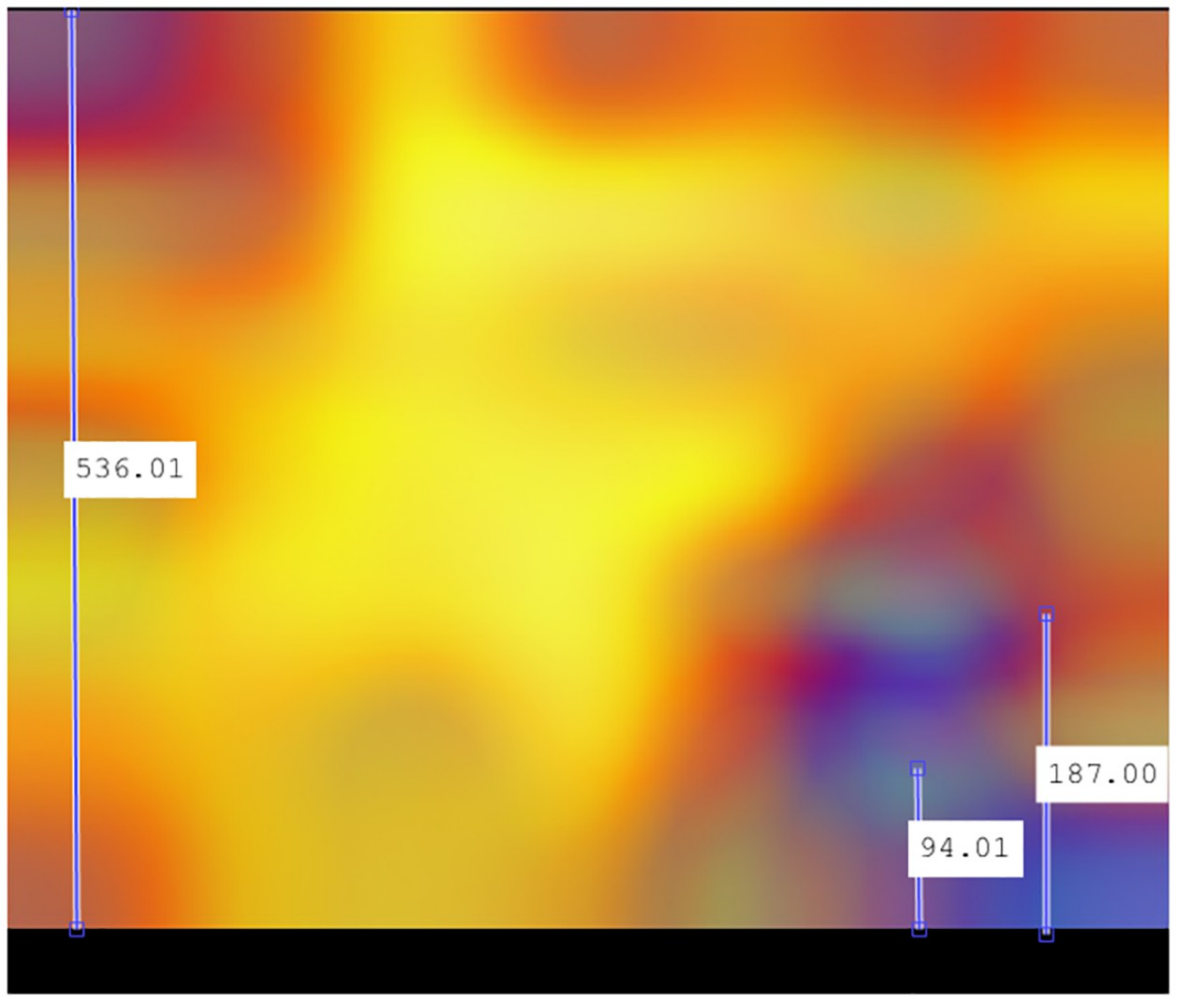
Vertical positioning of the epicenters of the main non-recombinant hot zones.

Figs 13–16 show the graphical analysis of the Total Hot Zones (Step 3) in Non-recombinants for 200 Epochs.

As seen in Fig 13, non-recombinant sequences do not exhibit a distinct hot zone, and the boundaries of hot zones are somewhat blurred. This phenomenon could be attributed to the greater diversity of sub-lineages and strains among non-recombinant variants, resulting in increased variability due to subsampling. As can be seen more clearly in Fig 14, subtle hotspots are hinted at around the Spike protein region. A potential third hotspot may exist towards the end of the genome, although its relevance appears to be less pronounced.

By direct extrapolation to the calculations shown in Fig 15, the epicenters of the main hot zones are situated at nucleotide positions between 23,341 and 23,362. The central position of the Spike protein (S) corresponds to nucleotide 23,473. Therefore, we can place the epicenters of both zones in the central region of the S protein.

By directly extrapolating from the calculations shown in Fig 16, regarding the vertical axis, considering its range is 0–0.5 Hz, the critical frequencies fall approximately at *f* = 1/12 and *f* = 1/6 respectively.

**Fig 17:**
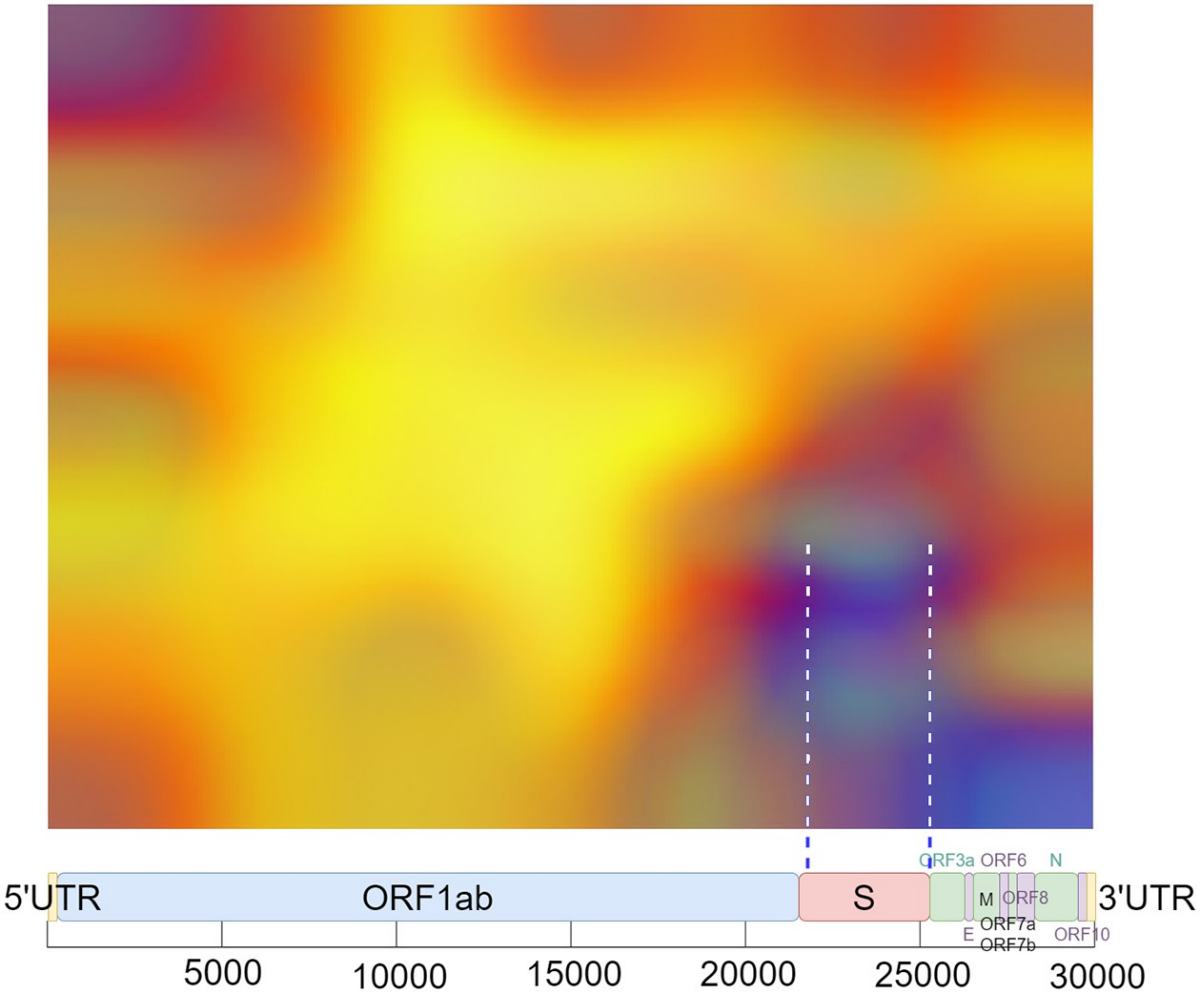
Localization of hot zones in non-recombinant sequences. Positioned in relation to a true-to-scale schematic representation of the composition of the SARS-CoV-2 genome. All notable proteins are appropriately marked.

In a comparison between the main hot zones using a representative scale of the SARS-CoV-2 genome, it becomes evident that the two primary hotspots are located in the vicinity of the Spike protein. See Fig 17.

Therefore, even though the areas are subtle, we can observe that the main decision regions are at S protein for the frequencies *f* = 1/12 and *f* = 1/6.

### 3.6 Analysis of recombinant results

**Fig 18:**
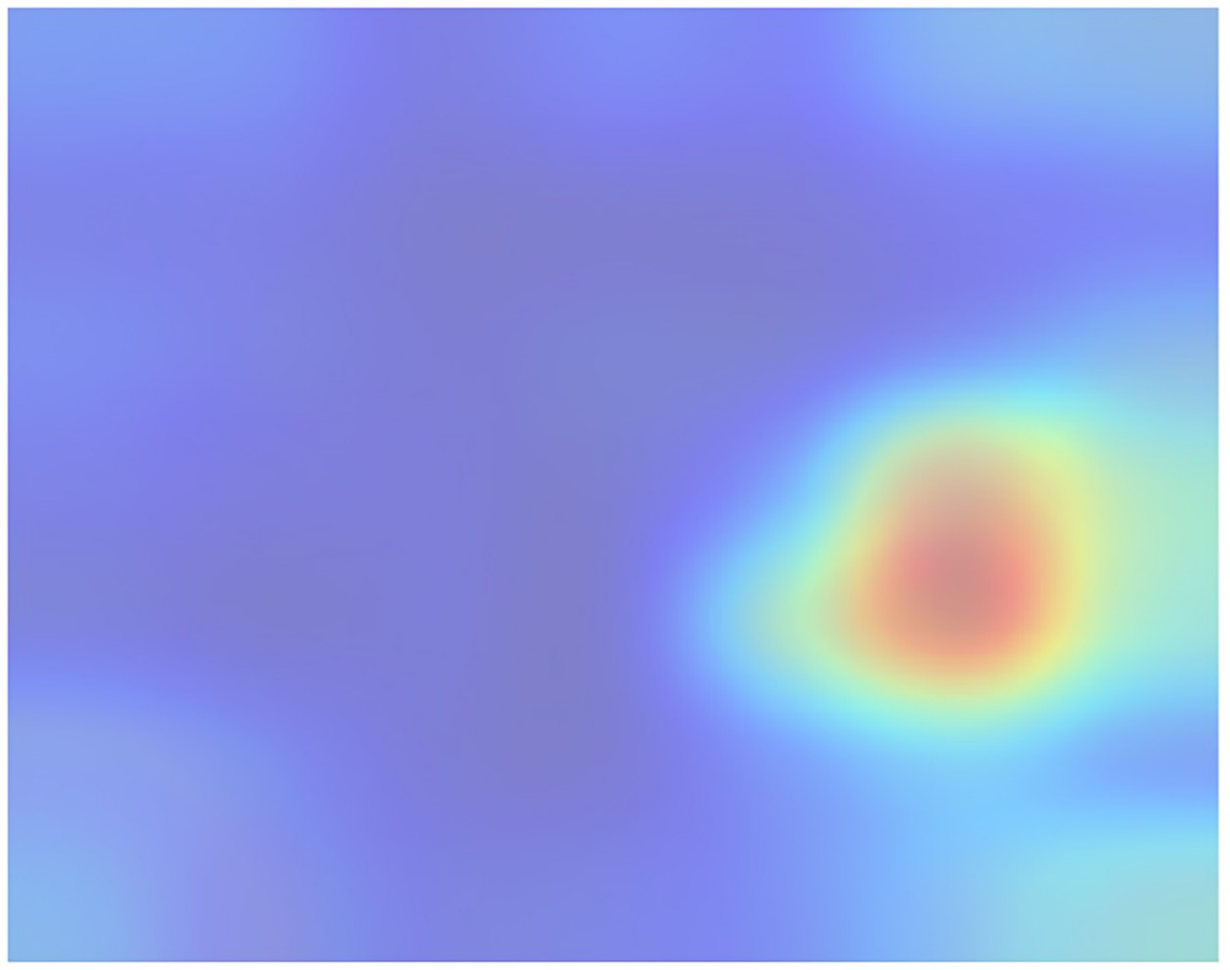
Recombinant total hot zones resulting from the 10 subsamplings for 200 Epochs.

**Fig 19:**
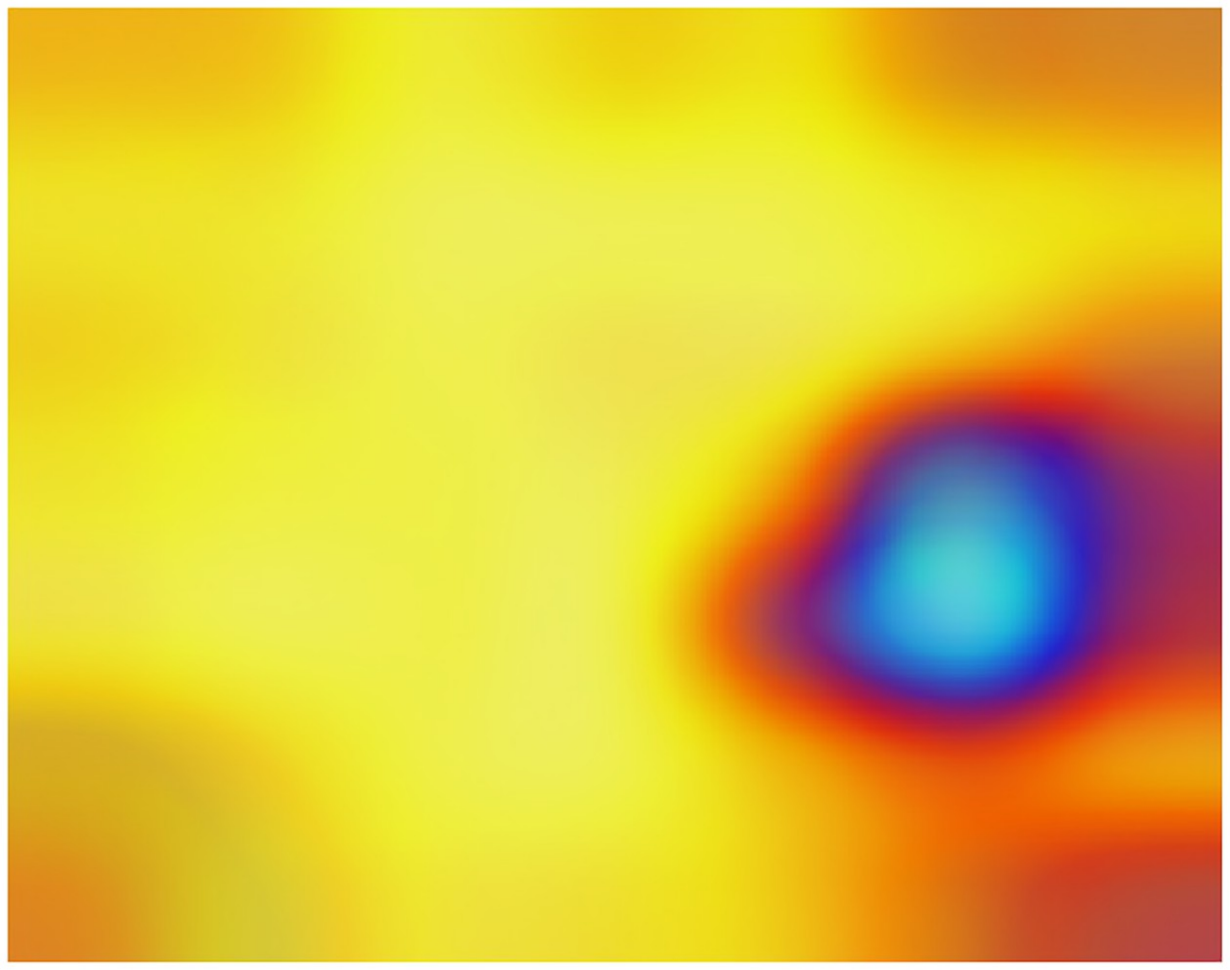
Image resulting from color scale enhancement for better recombinant hot zone identification.

**Fig 20:**
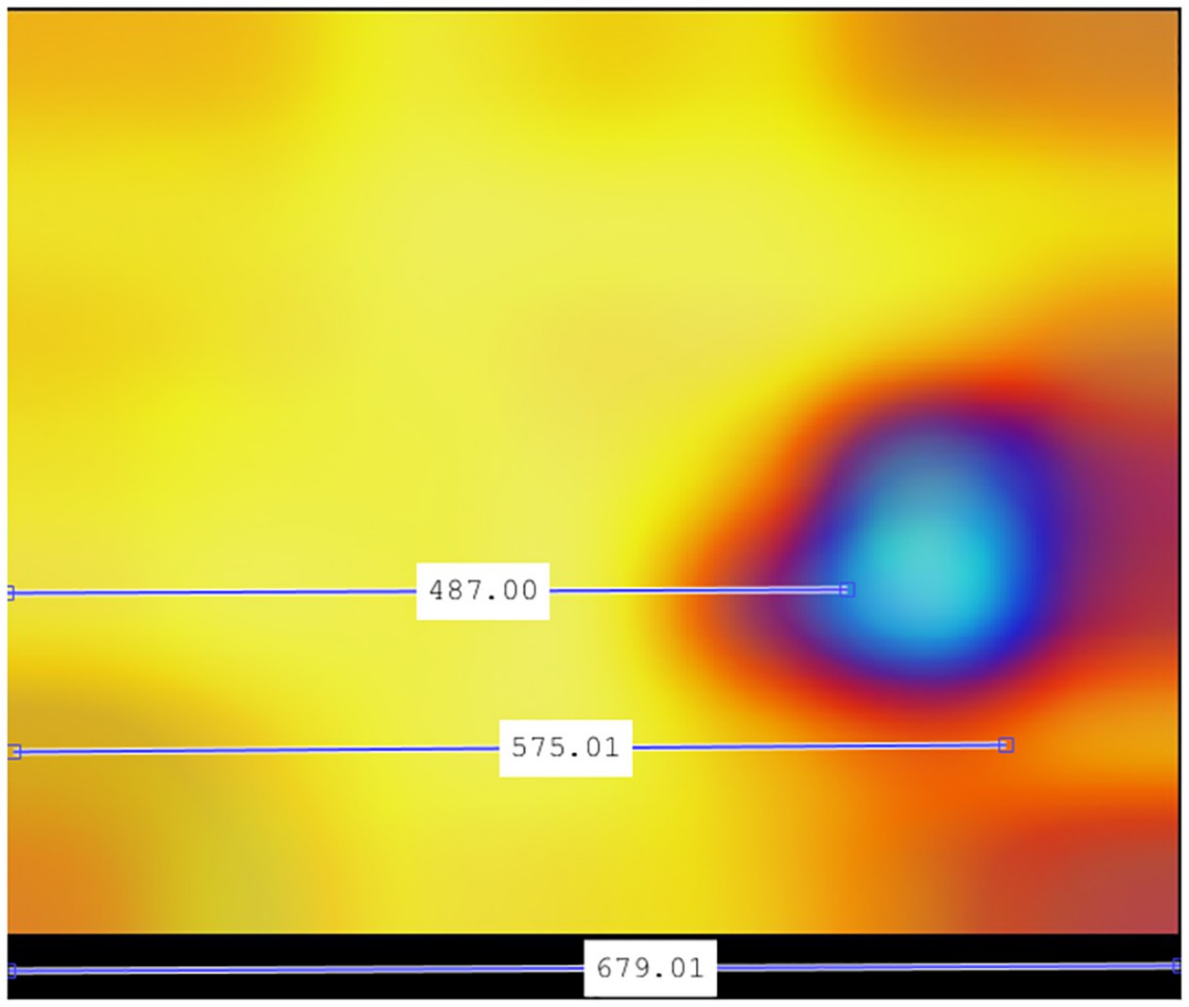
Horizontal positioning of the epicenters of the main recombinant hot zones.

**Fig 21:**
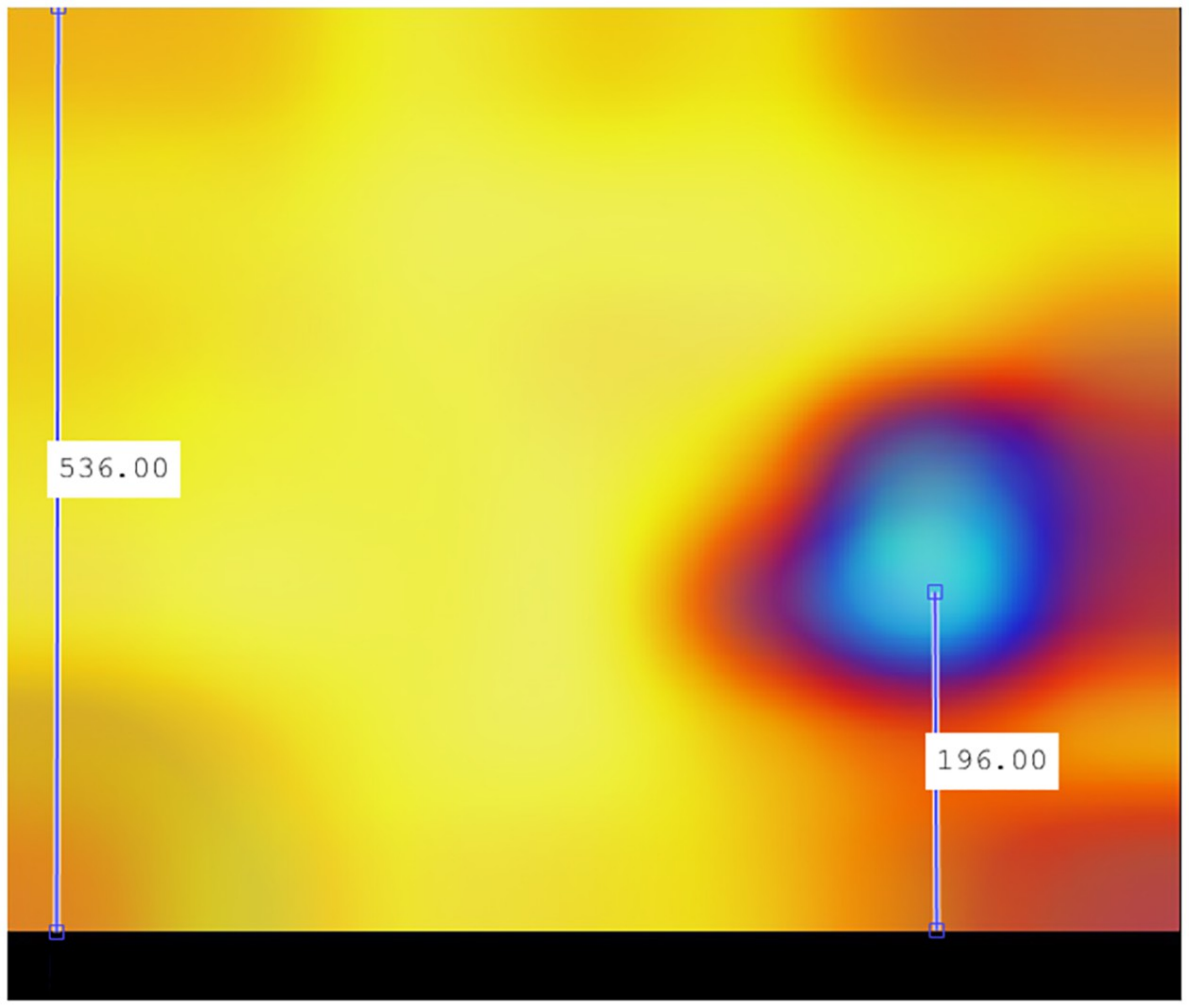
Vertical positioning of the epicenters of the main recombinant hot zones.

In the case of recombinant SARS-CoV-2 sequences, the main hot zone, where the CNN looks to detect the recombinant feature, is clearly delineated. Figs 18–21 compile the graphical analysis of the Total Hot Zones (Step 3) in Recombinants for 200 Epochs.

The sharpness of the total hot zone shown in Figs 18 and 19 denotes a prevalence of this hot spot across all subsamplings for 200 epochs.

After measuring the boundaries, when extrapolating to the length of the SARS-CoV-2 genome (29,903 nts.), the hot zone that determines a SARS-CoV-2 sequence to be recombinant is located roughly between positions 21,448 and 25,323 (see Fig 20). This location almost coincides with the position of the Spike protein (from approximately nucleotide 21,550 to roughly 25,400).

Regarding the vertical axis, whose total range is 0.5 Hz, as interpreted from the result in Fig 21, the preliminary identification of the epicenter of these total hot zones is located around 0.183 Hz, that is, around *f* = 1/6.

**Fig 22:**
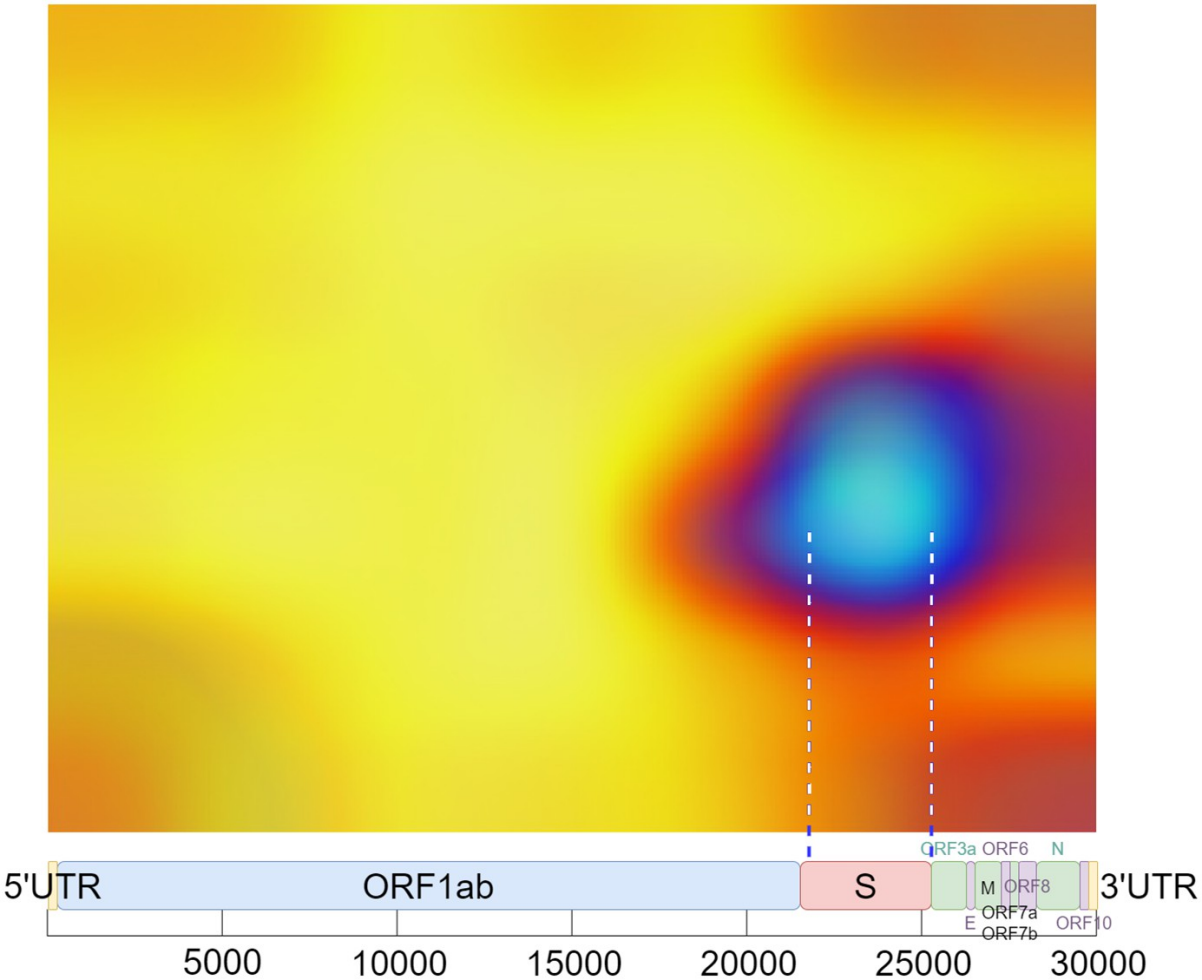
Localization of hot zones in recombinant sequences. Similarly to non-recombinant sequences, the main hot zones (Step 3) are positioned in relation to a scaled schematic representation of the SARS-CoV-2 genome.

Despite Grad-CAM’s imprecise interpretability, and considering that the location of each protein may vary depending on the variant and inherent sequence variability, the hot zone closely aligns enough to infer that the neural network is focusing on the S protein to identify the recombinant feature within the sequence. See Fig 22.

Using our methodology, we determined that the main hot zone is clearly located in the S protein. The use of this methodology allowed us to pinpoint the areas of the genomic spectrogram image where the pre-trained CNN “looks” to classify a sequence as recombinant or non-recombinant.

In coronaviruses, multiple genetic recombination events occurred in the S protein [59]. This phenomenon also happened in SARS-CoV-2 itself [60, 61].

At this point, we must make a distinction between the results obtained in our research and the fact that genetic recombination occurs in the S protein. In the research conducted on HIV-1 [45], the mathematical signature embedded in the genome that caused the pre-trained CNN to classify a sequence as recombinant or non-recombinant, was predominantly located in areas near the LTRs at a frequency of *f* = 1/3, regardless of the genomic regions where genetic recombination actually occurs between the different pure subtypes of HIV-1.

In the case of SARS-CoV-2, the location of this mathematical signature was detected in the same region where multiple genetic recombination events occurred. That is, the S protein.

The coincidence in location between the mathematical pattern, the mathematical signature detected by the pre-trained CNN, and the fact that the S protein is where abundant genetic recombination events occurred in SARS-CoV-2 should be studied in future investigations to determine if there is any relationship between these factors, as well as to unravel the significance of this phenomenon from a biological perspective.

Considering that the y-axis range is 0–0.5 Hz, this equates to a frequency of 0.18 Hz. Given the limited accuracy of Grad-CAM and the additive errors in the successive mathematical transformations performed in calculating the total hot zones, it is not unreasonable to consider the vertical epicenter at frequencies close to *f* = 1/6. To perform the most reliable verification of the epicenter frequency, we measured it in one of the experiments where this area is depicted most prominently: 200 Epochs in subsampling 06.

**Fig 23:**
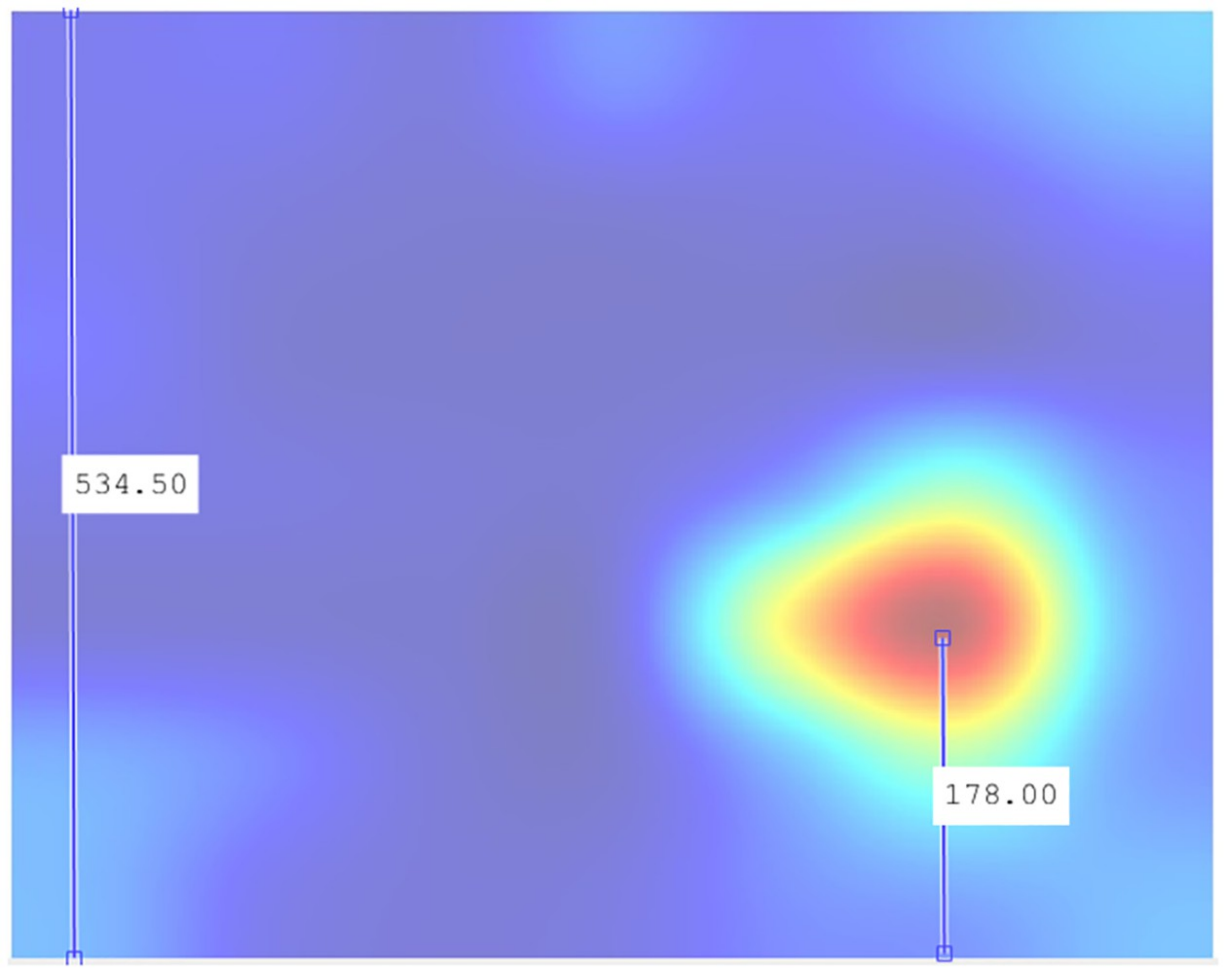
Vertical positioning at 200 Epochs configuration subsampling 06. Indeed, given that the total length of the y-axis is 534.5 points, with a range of 0–0.5 Hz, the epicenter’s position at 178 points precisely corresponds to 0.167, a value aligning with *f* = 1/6 in the sharpest case across the entire test bench.

Fig 23 displays the measurement obtained precisely at the epicenter of the hot zone. This figure underwent fewer mathematical transformations. Indeed, the vertical epicenter of the hot zone is located at frequencies close to *f* = 1/6, so it is a plausible hypothesis to consider this frequency as influential in determining the recombinant feature.

## 4 Conclusions

Using genomic spectrograms with 10 random subsamplings to address the disparity in size between non-recombinants and recombinants, we designed a test bench to elucidate the optimal hyperparameter configurations. We applied transfer learning in 2 phases using a pre-trained VGG-16 model on the ImageNet dataset. Phase 1 was focused on HIV-1 genomic spectrograms, and Phase 2 on those of SARS-CoV-2. All of this with the goal of detecting the recombinant feature of a SARS-CoV-2 genomic sequence. Subsequently, we applied the Grad-CAM interpretability tool in 3 steps to identify the hot zones (where the CNN looks for classification) in each sequence, in each subsample for every configuration, and in total in each configuration. We applied image processing techniques to enhance the localization of the hot zones. These 3 steps involve not only the mere application of Grad-CAM but also the mathematical processing of its results to extrapolate the obtained outcomes. The image processing techniques used allowed us to delineate the relevant areas for the recombinant feature as clearly as possible.

We obtained consistent and well-defined results in each category. In the case of SARS-CoV-2, the spike protein emerges as a determinant in both recombinant and non-recombinant categories.

The evident significance of the S protein in identifying the recombinant feature in SARS-CoV-2 aligns with the excellent research conducted by Nikolaidis et al. [59]. They uncovered multiple instances of double crossover genetic recombination events across various CoVs, and interestingly, the majority of these events are precisely located within this protein. Therefore, our work in a way reinforces their results by means of a different approach.

In the case of the non-recombinants, the hot zones (Step 3) are more diffuse, although they appear to pivot around the area of the spike protein within the frequency range of *f* = 1/12 and *f* = 1/6.

Nevertheless, the clarity of the main hot zones in Steps 2 and 3 is particularly striking in the case of recombinant sequences. A region corresponding to the Spike protein is clearly elucidated, at an approximate frequency of *f* = 1/6.

By utilizing Deep Learning tools, with their high potential in pattern recognition in images [62, 63], we were able to identify the determinant regions in the recombinant feature of genomic spectrograms of SARS-CoV-2. Achieving high test accuracy and robust, distinguishable hot zones in both categories.

## 5 Future research

In summary, we detected a mathematical signature that characterizes a genomic sequence of SARS-CoV-2 as recombinant. This signature is located in the S protein, with its epicenter at a frequency of *f* = 1/6. Consequently, the location of this mathematical signature is related to a nucleotide periodicity of 6, meaning that in the arithmetic series, every 6 nucleotides (two triplets) in S may encode crucial information related to the recombinant feature in SARS-CoV-2.

We know where the CNN looks to classify a SARS-CoV-2 sequence as recombinant. Now, we want to understand what it sees. What is the mathematical pattern embedded in the frequency spectrum of the genome of the Spike protein that causes a sequence to be classified as recombinant?

Our future research should focus on determining not only the formulation of this mathematical signature embedded in the genome but also its biological significance.

Another interesting line of research would be to determine the relationships between the dispersion detected in the hot zones in the non-recombinant category with the abundance and phylogenetic diversity of the set of non-recombinant variants in SARS-CoV-2.

In light of the results obtained, the identification of mathematical signatures in the virus genome through genomic spectrogram analysis opens up new avenues to investigate potential functions associated with these mathematical patterns.

## Supporting information

S1 Appendix Complete results per subsampling. (ZIP)

S2 Appendix Complete results per number of epochs. (ZIP)
