# Supplementary material for: Classification of SARS-CoV-2 sequences as recombinants via a pre-trained CNN and identification of a mathematical signature relative to recombinant feature at Spike, via interpretability
Source: PLoS One. 2024 Aug 26;19(8):e0309391. doi: 10.1371/journal.pone.0309391 (PMC11346643; doi:10.1371/journal.pone.0309391)
Supplement: S1 Appendix — (ZIP) [file pone.0309391.s001.zip › S1_appendix.pdf]

S1 Appendix. Complete results per subsampling

The results for each subsampling are presented in the following tables. The rows highlighted in green correspond to configurations with the highest test accuracy and a better balance between both categories.

In all cases, Learning Rate is 0.0001 and Batch Size is 52.

Table 1: Results in Subsampling 01 by the number of epochs. The configurations with 150 and 200 Epochs share optimal accuracy percentages in both categories. They exhibit the highest Test Accuracy values and the minimum inter-category SD. With the exception of the 10 and 25 Epoch configurations, which perform poorly compared to the other configurations, the rest demonstrate good accuracy rates.

SUBSAMPLING 01					
**Epochs**	**AUC**	**Validation Accuracy**	**Test Accuracy**	**Confusion Matrix**	**Inter Category SD**
10	0.9071	86.26%	84.03%	257 – 56<br>44 – 269	8.49
25	0.9529	87.54%	83.23%	294 – 19<br>86 – 227	47.38
50	0.9735	93.29%	93.13%	290 – 23<br>20 – 293	2.12
75	0.9769	91.37%	93.77%	287 – 26<br>13 – 300	9.19
100	0.9786	94.41%	94.89%	301 – 12<br>20 – 293	5.66
150	0.9818	94.25%	96.01%	302 – 11<br>14 – 299	2.12
200	0.9823	94.57%	96.01%	302 – 11<br>14 – 299	2.12
250	0.9830	94.41%	95.37%	301 – 12<br>17 – 296	3.54

Table 2: Results in Subsampling 02 by the number of epochs. The highest test accuracy values are achieved at 150 and 250 Epochs, both exceeding 95%. While the 150 Epoch configuration is slightly less balanced than the 250 Epoch one, we consider both configurations as optimal, with only 26 and 28 total errors in the test set.

**SUBSAMPLING 02**					
**Epochs**	**AUC**	**Validation Accuracy**	**Test Accuracy**	**Confusion Matrix**	**Inter Category SD**
10	0.8941	81.95%	80.19%	213 – 100<br>24 – 289	53.74
25	0.9371	89.94%	85.46%	289 – 24<br>67 – 246	30.41
50	0.9714	94.41%	92.49%	279 – 34<br>13 – 300	14.85
75	0.9684	93.77%	92.33%	303 – 10<br>38 – 275	19.80
100	0.9814	94.73%	94.89%	298 – 15<br>17 – 296	1.41
150	0.9806	94.57%	95.85%	303 – 10<br>16 – 297	4.24
200	0.9784	94.73%	94.57%	293 – 20<br>14 – 299	4.24
250	0.9796	95.05%	95.53%	298 – 15<br>13 – 300	1.41

Table 3: Results in Subsampling 03 by the number of epochs. The 200 Epoch configuration exhibits the optimal test accuracy value with a high balance between the two categories (Inter-Category SD lower than 1%). These results are significantly better than those of the remaining configurations.

**SUBSAMPLING 03**					
**Epochs**	**AUC**	**Validation Accuracy**	**Test Accuracy**	**Confusion Matrix**	**Inter Category SD**
10	0.9224	84.66%	82.11%	222 – 91<br>21 – 292	49.50
25	0.9692	89.62%	90.58%	277 – 36<br>23 – 290	9.19
50	0.9745	93.93%	92.33%	297 – 16<br>32 – 281	11.31
75	0.9696	93.77%	91.21%	295 – 18<br>37 – 276	13.44
100	0.9812	95.37%	94.57%	299 – 14<br>20 – 293	4.24
150	0.9807	94.89%	94.57%	301 – 12<br>22 – 291	7.07
200	0.9824	95.21%	94.73%	297 – 16<br>17 – 296	0.71
250	0.9805	94.41%	94.09%	301 – 12<br>25 – 288	9.19

Table 4: Results in Subsampling 04 by the number of epochs. The 200 Epoch configuration achieves the highest test accuracy value. Although the Inter-Category SD is slightly higher than at 150 Epochs, the total of 28 errors compared to 31 errors in the case of 150 Epochs lead us to select this configuration as the best for this subsampling.

**SUBSAMPLING 04**					
**Epochs**	**AUC**	**Validation Accuracy**	**Test Accuracy**	**Confusion Matrix**	**Inter Category SD**
10	0.9039	88.18%	83.23%	246 – 67<br>38 – 275	20.51
25	0.9678	92.97%	89.78%	286 – 27<br>37 – 276	7.07
50	0.9784	94.57%	93.45%	297 – 16<br>25 – 288	6.36
75	0.9786	94.57%	93.77%	295 – 18<br>21 – 292	2.12
100	0.9769	94.25%	92.49%	294 – 19<br>28 – 285	6.36
150	0.9811	95.69%	95.05%	302 – 11<br>20 – 293	6.36
200	0.9808	96.17%	95.53%	304 – 9<br>19 – 294	7.07
250	0.9790	95.69%	94.57%	303 – 10<br>24 – 289	9.90

Table 5: Results in Subsampling 05 by the number of epochs. The configurations of 200 and 250 Epochs share the same test accuracy value, with a slight penalty in the inter-category standard deviation for 250 Epochs compared to 200 Epochs.

**SUBSAMPLING 05**					
**Epochs**	**AUC**	**Validation Accuracy**	**Test Accuracy**	**Confusion Matrix**	**Inter Category SD**
10	0.8755	84.35%	79.23%	254 – 59<br>71 – 242	8.49
25	0.9481	91.53%	88.18%	268 – 45<br>29 – 284	11.31
50	0.9486	92.49%	85.30%	297 – 16<br>76 – 237	42.43
75	0.9680	94.73%	92.17%	285 – 28<br>21 – 292	4.95
100	0.9613	93.93%	90.58%	291 – 22<br>37 – 276	10.61
150	0.9722	94.73%	92.97%	293 – 20<br>24 – 289	2.83
200	0.9698	95.53%	93.13%	294 – 19<br>24 – 289	3.54
250	0.9732	95.21%	93.13%	288 – 25<br>18 – 295	4.95

Table 6: Results in Subsampling 06 by the number of epochs. The 200 Epoch configuration is clearly the optimal one compared to the other configurations, both in terms of test accuracy values and, above all, Inter-Category SD.

**SUBSAMPLING 06**					
**Epochs**	**AUC**	**Validation Accuracy**	**Test Accuracy**	**Confusion Matrix**	**Inter Category SD**
10	0.8746	85.30%	79.71%	257 – 56<br>71 – 242	10.61
25	0.9390	89.30%	87.22%	277 – 36<br>44 – 269	5.66
50	0.9644	92.97%	91.69%	290 – 23<br>29 – 284	4.24
75	0.9665	93.93%	92.17%	289 – 24<br>25 – 288	0.71
100	0.9737	94.41%	94.25%	299 – 14<br>22 – 291	5.66
150	0.9744	93.93%	93.93%	304 – 9<br>29 – 284	14.14
200	0.9782	94.57%	94.57%	297 – 16<br>18 – 295	1.41
250	0.9788	94.73%	94.25%	300 – 13<br>23 – 290	7.07

Table 7: Results in Subsampling 07 by the number of epochs. The 150 and 200 configurations are the only ones that surpass 95% test accuracy. Although the Inter-Category SD is high in both configurations, especially when compared to 100 Epochs, we consider that, in this subsampling, even though the accuracy between both categories is imbalanced, these configurations are the most stable in overall performance.

**SUBSAMPLING 07**					
**Epochs**	**AUC**	**Validation Accuracy**	**Test Accuracy**	**Confusion Matrix**	**Inter Category SD**
10	0.9108	84.50%	83.55%	274 – 39<br>64 – 249	17.68
25	0.9589	92.17%	87.50%	299 – 14<br>63 – 250	34.65
50	0.9795	93.93%	94.57%	301 – 12<br>22 – 291	7.07
75	0.9793	93.93%	92.49%	311 – 2<br>45 – 268	30.41
100	0.9813	94.89%	92.97%	293 – 20<br>24 – 289	2.83
150	0.9824	94.73%	95.37%	309 – 4<br>25 – 288	14.85
200	0.9806	94.73%	95.21%	305 – 8<br>22 – 291	9.90
250	0.9806	94.57%	93.77%	297 – 16<br>23 – 290	4.95

Table 8: Results in Subsampling 08 by the number of epochs. In this case, we consider the 150 and 200 Epoch configurations as the optimal ones due to their total hit rate in the test set, which is slightly superior to the 100 Epoch configuration, even though the Inter-Category SD is slightly lower.

**SUBSAMPLING 08**					
**Epochs**	**AUC**	**Validation Accuracy**	**Test Accuracy**	**Confusion Matrix**	**Inter Category SD**
10	0.9136	84.82%	81.95%	235 – 78<br>35 – 278	30.41
25	0.9461	85.78%	81.63%	306 – 7<br>108 – 205	71.42
50	0.9735	93.61%	93.13%	290 – 23<br>20 – 293	2.12
75	0.9714	93.93%	93.77%	297 – 16<br>23 – 290	4.95
100	0.9682	92.81%	90.26%	272 – 41<br>20 – 293	14.85
150	0.9769	91.05%	91.69%	275 – 38<br>14 – 299	16.97
200	0.9754	91.69%	91.69%	275 – 38<br>14 – 299	16.97
250	0.9739	93.93%	93.29%	292 – 21<br>21 – 292	0.00

Table 9: Results in Subsampling 09 by the number of epochs. In this case, we have chosen the 100 Epoch configuration as the optimal one due to its highest test accuracy value and lowest Inter-Category SD.

**SUBSAMPLING 09**					
**Epochs**	**AUC**	**Validation Accuracy**	**Test Accuracy**	**Confusion Matrix**	**Inter Category SD**
10	0.9324	83.55%	86.58%	266 – 47<br>37 – 276	7.07
25	0.9629	90.58%	89.46%	295 – 18<br>48 – 265	21.21
50	0.9735	86.10%	93.13%	290 – 23<br>20 – 293	2.12
75	0.9804	93.45%	94.73%	301 – 12<br>21 – 292	6.36
100	0.9842	94.89%	96.17%	305 – 8<br>16 – 297	5.66
150	0.9843	94.89%	95.69%	307 – 6<br>21 – 292	10.61
200	0.9845	94.89%	95.53%	307 – 6<br>22 – 291	11.31
250	0.9831	94.73%	95.21%	304 – 9<br>21 – 292	8.49

Table 10: Results in Subsampling 10 by the number of epochs. The optimal configuration is clearly 200 Epochs, as it is the only one to exceed 96% test accuracy with low levels of Inter-Category SD.

**SUBSAMPLING 10**					
**Epochs**	**AUC**	**Validation Accuracy**	**Test Accuracy**	**Confusion Matrix**	**Inter Category SD**
10	0.8786	83.87%	78.91%	268 – 45<br>87 – 226	29.70
25	0.9525	89.62%	84.98%	305 – 8<br>86 – 227	55.15
50	0.9590	92.17%	92.33%	286 – 27<br>21 – 292	4.24
75	0.9703	92.33%	93.93%	295 – 18<br>20 – 293	1.41
100	0.9739	93.29%	94.41%	303 – 10<br>25 – 288	10.61
150	0.9726	93.45%	94.89%	304 – 9<br>23 – 290	9.90
200	0.9728	93.45%	95.05%	302 – 11<br>20 – 293	6.36
250	0.9729	93.77%	95.05%	302 – 11<br>20 – 293	6.36
