# Supplementary material for: Classification of SARS-CoV-2 sequences as recombinants via a pre-trained CNN and identification of a mathematical signature relative to recombinant feature at Spike, via interpretability
Source: PLoS One. 2024 Aug 26;19(8):e0309391. doi: 10.1371/journal.pone.0309391 (PMC11346643; doi:10.1371/journal.pone.0309391)
Supplement: S2 Appendix — (ZIP) [file pone.0309391.s002.zip › S2_appendix.pdf]

S2 Appendix. Complete results per number of epochs

Table 1: Results at 10 Epochs. Comparing to the entire test dataset (313 genomic spectrograms per category), the average test accuracy can be improved. The hit rate is low, and the inter-category standard deviation is high, indicating an excessive imbalance between the accuracy in both categories. This configuration likely suffers from a lack of training.

**10 EPOCHS**				
**SUBSAMPLING**	**Non-rec. Test Acc.**	**Non-rec. Test Err.**	**Rec. Test Acc.**	**Rec. Test Err.**
SUB_01	257	56	269	44
SUB_02	213	100	289	24
SUB_03	222	91	292	21
SUB_04	246	67	275	38
SUB_05	254	59	242	71
SUB_06	257	56	242	71
SUB_07	274	39	249	64
SUB_08	235	78	278	35
SUB_09	266	47	276	37
SUB_10	268	45	226	87
**Avg.**	**249.2**		**263.8**	
**SD**	**20.16**		**22.44**	
**Inter Category SD = 10.32**				

Table 2: Results at 25 Epochs. Similar to the previous case, this configuration is in need of improvement in terms of accuracy in both categories. It is particularly striking that there is a high imbalance between the accuracy of recombinants (which is also only 81%) and non-recombinants, exceeding even 25 points. This configuration lacks sufficient training and is therefore not suitable.

**25 EPOCHS**				
**SUBSAMPLING**	**Non-rec. Test Acc.**	**Non-rec. Test Err.**	**Rec. Test Acc.**	**Rec. Test Err.**
SUB_01	294	19	227	86
SUB_02	289	24	246	67
SUB_03	277	36	290	23
SUB_04	286	27	276	37
SUB_05	268	45	284	29
SUB_06	277	36	269	44
SUB_07	299	14	250	63
SUB_08	306	7	205	108
SUB_09	295	18	265	48
SUB_10	305	8	227	86
**Avg.**	**289.6**		**253.9**	
**SD**	**12.65**		**27.82**	
**Inter Category SD = 25.24**				

Table 3: Results at 50 Epochs. The average test accuracy values are better compared to previous configurations in both categories, as well as the balance between both values. However, when compared to the subsequent configurations, we observe some potential for improvement by increasing the number of epochs.

**50 EPOCHS**				
**SUBSAMPLING**	**Non-rec. Test Acc.**	**Non-rec. Test Err.**	**Rec. Test Acc.**	**Rec. Test Err.**
SUB_01	290	23	293	20
SUB_02	279	34	300	13
SUB_03	297	16	281	32
SUB_04	297	16	288	25
SUB_05	297	16	237	76
SUB_06	290	23	284	29
SUB_07	301	12	291	22
SUB_08	290	23	293	20
SUB_09	290	23	293	20
SUB_10	286	27	292	21
**Avg.**	**291.7**		**285.2**	
**SD**	**6.46**		**17.74**	
**Inter Category SD = 4.60**				

Table 4: Results at 75 Epochs. No significant improvements compared to 50 Epochs. While there are slight enhancements in test accuracy values in both categories, the inter-category standard deviation is slightly higher.

**75 EPOCHS**				
**SUBSAMPLING**	**Non-rec. Test Acc.**	**Non-rec. Test Err.**	**Rec. Test Acc.**	**Rec. Test Err.**
SUB_01	287	26	300	13
SUB_02	303	10	275	38
SUB_03	295	18	276	37
SUB_04	295	18	292	21
SUB_05	285	28	292	21
SUB_06	289	24	288	25
SUB_07	311	2	268	45
SUB_08	297	16	290	23
SUB_09	301	12	292	21
SUB_10	295	18	293	20
**Avg.**	**295.8**		**286.6**	
**SD**	**7.84**		**10.08**	
**Inter Category SD = 6.51**				

Table 5: Results at 100 Epochs. Both test accuracy values surpass 290 correct predictions (over 92%), and the inter-category standard deviation drops below 4 points for the first time. This configuration may be sufficient, although there is potential for improvement.

**100 EPOCHS**				
**SUBSAMPLING**	**Non-rec. Test Acc.**	**Non-rec. Test Err.**	**Rec. Test Acc.**	**Rec. Test Err.**
SUB_01	301	12	293	20
SUB_02	298	15	296	17
SUB_03	299	14	293	20
SUB_04	294	19	285	28
SUB_05	291	22	276	37
SUB_06	299	14	291	22
SUB_07	293	20	289	24
SUB_08	272	41	293	20
SUB_09	305	8	297	16
SUB_10	303	10	288	25
**Avg.**	**295.5**		**290.1**	
**SD**	**9.36**		**6.14**	
**Inter Category SD = 3.82**				

Table 6: Results at 150 Epochs. This configuration exhibits the highest test accuracy value in non-recombinants. However, it is slightly penalized by the imbalance with respect to the recombinants, where it performs well but is outperformed by the 200 Epoch configuration.

**150 EPOCHS**				
**SUBSAMPLING**	**Non-rec. Test Acc.**	**Non-rec. Test Err.**	**Rec. Test Acc.**	**Rec. Test Err.**
SUB_01	302	11	299	14
SUB_02	303	10	297	16
SUB_03	301	12	291	22
SUB_04	302	11	293	20
SUB_05	293	20	289	24
SUB_06	304	9	284	29
SUB_07	309	4	288	25
SUB_08	275	38	299	14
SUB_09	307	6	292	21
SUB_10	304	9	290	23
**Avg.**	**300.0**		**292.2**	
**SD**	**9.74**		**4.92**	
Inter Category SD = 5.52				

Table 7: Results at 200 Epochs. Chosen as the optimal configuration due to its high accuracy rates in both categories and its optimal balance between both test accuracy.

**200 EPOCHS**				
**SUBSAMPLING**	**Non-rec. Test Acc.**	**Non-rec. Test Err.**	**Rec. Test Acc.**	**Rec. Test Err.**
SUB_01	302	11	299	14
SUB_02	293	20	299	14
SUB_03	297	16	296	17
SUB_04	304	9	294	19
SUB_05	294	19	289	24
SUB_06	297	16	295	18
SUB_07	305	8	291	22
SUB_08	275	38	299	14
SUB_09	307	6	291	22
SUB_10	302	11	293	20
**Avg.**	**297.6**		**294.6**	
**SD**	**9.24**		**3.66**	
**Inter Category SD = 2.12**				

Table 8: Results at 250 Epochs. The hit rate in recombinants decreases slightly compared to the 200 Epoch configuration. Additionally, the inter-category standard deviation is slightly higher. While it may not be the optimal configuration, the obtained results are positive.

**250 EPOCHS**				
**SUBSAMPLING**	**Non-rec. Test Acc.**	**Non-rec. Test Err.**	**Rec. Test Acc.**	**Rec. Test Err.**
SUB_01	301	12	296	17
SUB_02	298	15	300	13
SUB_03	301	12	288	25
SUB_04	303	10	289	24
SUB_05	288	25	295	18
SUB_06	300	13	290	23
SUB_07	297	16	290	23
SUB_08	292	21	292	21
SUB_09	304	9	292	21
SUB_10	302	11	293	20
**Avg.**	**298.6**		**292.5**	
**SD**	**5.08**		**3.66**	
**Inter Category SD = 4.31**				
